# Low‐Frequency Noise and Stability in Electrolyte‐Gated Nanoscale Silicon Field‐Effect Transistor Sensors

**DOI:** 10.1002/smtd.71112

**Published:** 2026-10-13

**Authors:** Yingtao Yu, Zheqiang Xu, Yihan Wang, Qitao Hu, Paul Solomon, Zhen Zhang

**Affiliations:** ^1^ Ångström Laboratory, Division of Solid‐State Electronics, Department of Electrical Engineering Uppsala University Uppsala Sweden; ^2^ Department of Radiology Stanford University Stanford California USA; ^3^ IBM Thomas J. Watson Research Center Yorktown Heights New York USA

**Keywords:** 1/*f* noise, limit‐of‐detection, low‐frequency noise, nanoscale silicon FET, Schottky‐junction gate, signal‐to‐noise, solid–liquid interface

## Abstract

Electrolyte‐gated nanoscale silicon field‐effect transistors (Si FETs), including silicon nanoribbon FETs (SiNRFETs) and nanowire FETs (SiNWFETs), have emerged as highly promising platforms for chemical and biological sensing. As these sensors move from proof‐of‐concept demonstrations toward quantitative assays, practical performance is increasingly governed not only by nominal sensitivity, but also by their signal‐to‐noise ratio (SNR) and baseline stability. Low‐frequency noise (LFN), typically arising from carrier‐number and mobility fluctuations in the solid‐state device, as well as reversible ion adsorption/desorption processes at the solid–liquid interfaces, is generally characterized by an approximately 1/*f*‐dependent power spectral density. It limits the minimum resolvable signal and thus determines the limit‐of‐detection (LoD). In parallel, slower nonstationary processes, including ion penetration and reference‐electrode drift, cause baseline instability that complicates quantitative sensing and array calibration. This review discusses LFN and instability in electrolyte‐gated nanoscale Si FET sensors as a coupled device‐interface‐readout problem. Mitigation strategies are also summarized. Representative examples illustrate how dominant noise sources can be identified and suppressed. Finally, we highlight the applications of low‐noise and low‐drift FET sensors. Next‐generation electrolyte‐gated nanoscale Si FET sensors require codesign of the transducer, sensing interface, electrolyte/reference environment, and front‐end electronics to achieve a low LoD and high detection accuracy.

## Graphical abstract

Electrolyte‐gated nanoscale silicon field‐effect transistor sensors provide exceptional charge sensitivity and direct electrical readout. However, their sensing accuracy is fundamentally limited by low‐frequency noise and baseline drift. This review examines the origins of noise and drift in these sensors, summarizes the key mitigation strategies, and highlights the importance of coordinated optimization across the transducer, sensing interface, electrolyte/electrode environment, and readout.

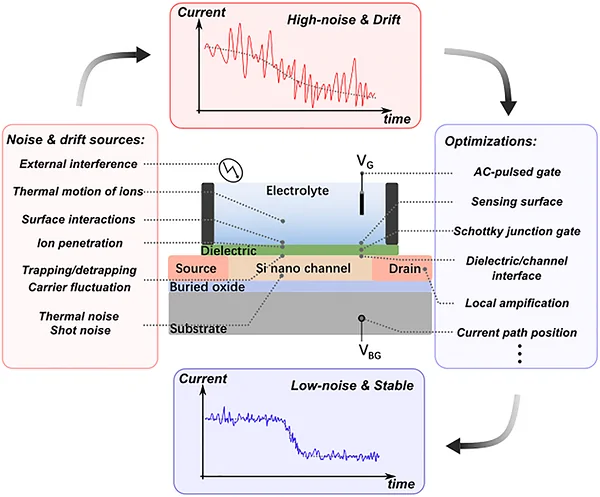

## 1 Introduction

Since the introduction of the ion‐sensitive field‐effect transistor (ISFET) by Bergveld [1, 2], electrolyte‐gated silicon field‐effect transistor (FET) sensors have evolved from planar pH‐sensitive transistors into a diverse family of integrated ion‐ and biomolecule‐sensing platforms, including extended‐gate ISFETs [3, 4, 5, 6], silicon nanoribbon FETs (SiNRFETs) [7, 8, 9, 10], silicon nanowire FETs (SiNWFETs) [11, 12, 13, 14], and large‐scale CMOS‐compatible ion‐imaging arrays [15, 16]. Device downscaling can enhance the response to localized or discrete surface charges and improve spatial resolution, but it does not universally increase the gate‐referred sensitivity to spatially uniform pH or ion signals. At the same time, the key performance metric has gradually shifted from sensitivity alone toward high signal‐to‐noise ratio (SNR) and high‐accuracy sensing [17]. This shift is driven by the need to resolve weaker biochemical signals in smaller sensing areas and higher‐density arrays, where reduced statistical averaging over carriers, traps, and interfacial sites increases the impact of low‐frequency noise (LFN) and drift. In addition, array‐level operation makes baseline stability, pixel‐to‐pixel variation, reference‐electrode stability, parasitic pickup, and readout‐chain noise increasingly important for quantitative sensing.

Here, Figure 1a [1, 11, 17, 18, 19, 20, 21, 22, 23, 24] places this review within a broader historical trajectory shaped by several research communities. Bergveld first introduced the ISFET in 1970 [1, 2], followed by the early analysis of ISFET noise by Haemmerli and coworkers [18]. Cui, Lieber, and coworkers subsequentially established silicon nanowires as highly sensitive chemical and biological transducers [11, 12]. Later, the groups of Schönenberger, Calame, and Reed subsequently advanced dual‐gate operation [19], noise characterization, and SNR‐oriented sensor design [17]. Later studies by Bedner and coworkers identified dominant device‐level 1/*f* noise sources [20]. Then our group established experimental and kinetic frameworks for solid–liquid interfacial noise. Our more recent Schottky‐junction‐gated and tri‐gated FET devices further reduced intrinsic transducer noise and ultimately achieved single‐charge‐resolved ion sensing on sub‐10‐nm SiNWFET devices. These milestones demonstrate that the evolution of ISFET and nanoscale Si FET sensing has involved not only device downscaling and integration, but also a progressive shift toward understanding and suppressing noise throughout the signal‐transduction chain. Excellent reviews are available for ISFET sensors and their applications [25, 26, 27]. Here, we focus specifically on the evolution of LFN understanding and mitigation in nanoscale Si FET sensors, with an emphasis on how device physics, sensing‐interface dynamics, and readout architecture jointly determine the practical SNR and limit‐of‐detection (LoD). Contributions from the broader community are reviewed together with selected examples from our previous work where they illustrate specific noise mechanisms or mitigation strategies.

**FIGURE 1:**
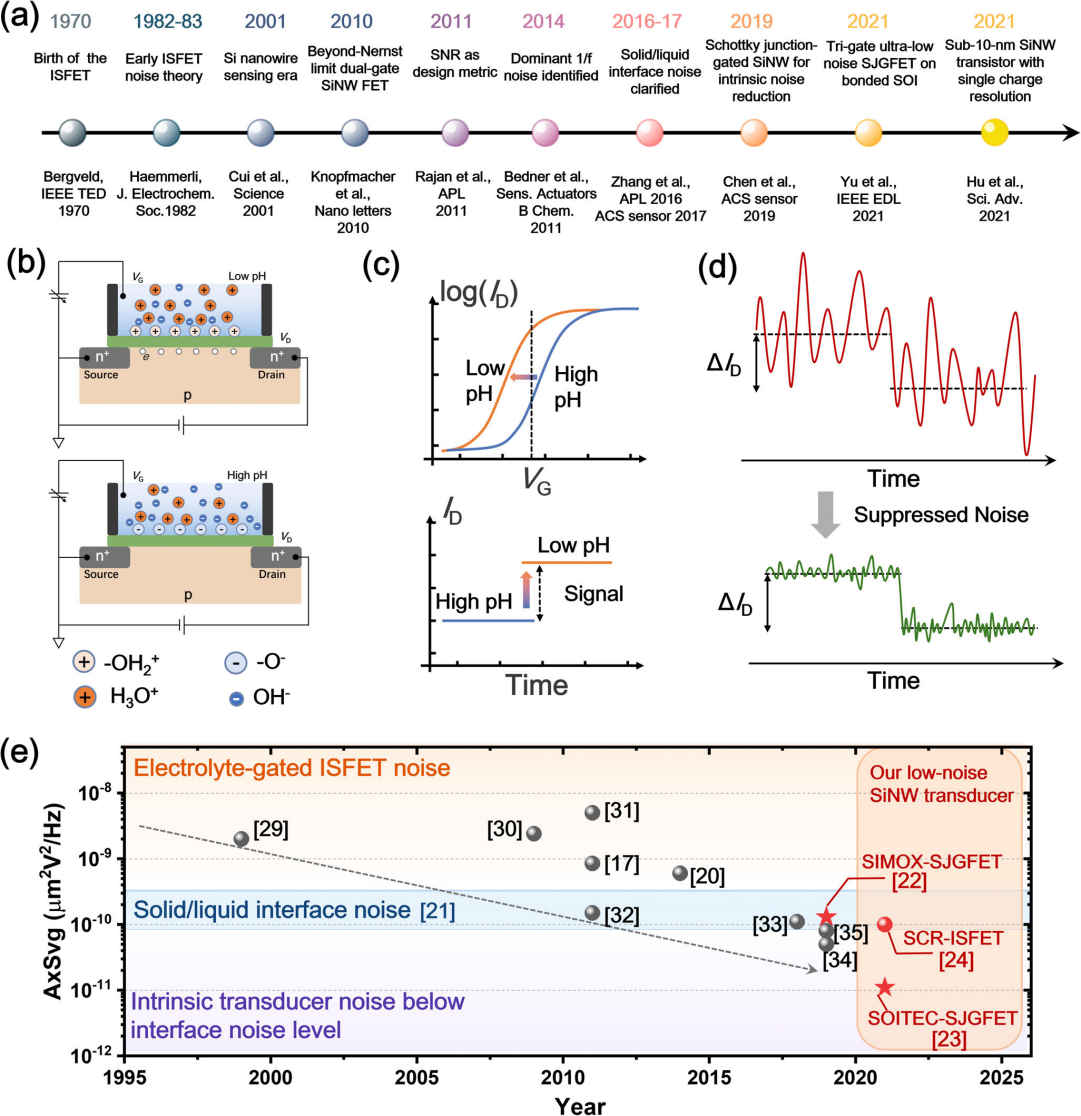
(a) Historical development of ISFETs and nanoscale Si FET sensors, together with representative milestones in LFN understanding and reduction, showing the evolution from sensitivity‐driven ISFET scaling to high‐SNR low‐noise nanoscale Si FET sensing. (b) Schematic illustration of pH‐dependent protonation and deprotonation at the oxide/electrolyte interface of an ISFET. (c) pH‐induced threshold‐voltage shift in the transfer characteristics (*I*_D_ vs *V*_G_) and corresponding *I*_D_ response under constant *V*_G_ sampling. (d) Conceptual comparison showing that high sensitivity alone is insufficient for quantitative sensing, suppression of equivalent input noise is required to improve SNR and effective resolution. (e) Noise roadmap [17, 20, 21, 22, 23, 24, 29, 30, 31, 32, 33, 34, 35] based on reported works. Area‐normalized gate‐referred voltage noise, *A*×*S*_vg_, referenced to 10 Hz, is used to enable comparison across devices with different geometries. The roadmap highlights the progressive reduction of transducer noise from conventional oxide‐gated ISFETs and SiNWFETs toward ultralow‐noise nanoscale Si FETs, reaching noise levels below the reported solid–liquid interfacial noise floor.

Despite differences in device geometry, sensing layer, and readout architecture, electrolyte‐gated FET sensors share a common transduction mechanism: chemical or biological activity at the solid–liquid interface is converted into a modulation of the channel conductance [28]. A pH‐sensitive ISFET provides the most representative example (Figure 1b). In low‐pH solutions, amphoteric hydroxyl groups on the oxide surface are protonated, forming positively charged surface sites. In high‐pH solutions, these groups are deprotonated, generating negatively charged surface sites. The resulting change in the surface charge of the sensing oxide modifies the potential distribution across the electrical double layer and shifts the effective gate potential coupled to the transistor channel. This surface potential modulation is observed electrically either as a threshold voltage shift in the transfer characteristics or as a drain current change during constant‐bias sampling (Figure 1c).

For many years, nanoscale FET sensor development has often followed a sensitivity‐driven scaling principle: reducing the channel dimensions can increase the surface‐to‐volume ratio and enhance the conductance response to localized surface charges or small‐volume ionic activity [36]. This benefit is most relevant for weak, spatially localized signals, such as low‐abundance biomolecular binding, single‐charge or interfacial‐event detection, and single‐cell or few‐cell metabolic sensing. By contrast, for spatially uniform pH or ion‐concentration measurements, the nominal sensitivity is mainly determined by the sensing layer, surface buffer effect, and electrical‐double‐layer (EDL) coupling and is not inherently enhanced by reducing the sensing area. Therefore, the effect of device geometry must be considered together with noise. Device scaling reduces the number of carriers in the channel, decreases the effective gate area, weakens statistical averaging over independent fluctuations, and increases the electrostatic impact of individual traps or surface‐charge fluctuations [37, 38]. For noise dominated by statistically independent traps or interfacial fluctuators, the gate‐referred voltage‐noise spectral density (*S*_vg_) approximately follows *S*_vg_ ∝ 1/*A*, where *A* is the effective active gate‐coupled area. This relation motivates the use of the area‐normalized metric *A*×*S*_vg_ for comparing devices with different geometries. Deviations from this scaling may occur when a few discrete traps dominate, or when contact, electrolyte‐interface, or readout noise contributes significantly. Consequently, downscaling can strengthen the coupling to localized signals and improve spatial resolution while simultaneously increasing the absolute input‐referred noise [20, 39, 40]. For spatially uniform signals, whose gate‐referred amplitude is approximately independent of sensing area, a larger device area can improve SNR through stronger statistical noise averaging. In contrast, for localized or discrete events, the enhanced signal coupling provided by a smaller device may compensate for, or outweigh, the accompanying increase in noise. The practical sensing performance is consequently determined not by sensitivity alone, but by the SNR [17, 41], which defines whether a given chemical signal can be resolved above the noise floor (Figure 1d). For a gate‐referred sensing signal, Δ*V*_sig_, the practical SNR is determined by the bandwidth‐integrated root mean square (RMS) gate‐referred noise, $V_{n,rms}={\left[\int_{f_{L}}^{f_{H}}S_{Vg}\left(f\right)df\right]}^{1/2}$, such that *SNR* = |Δ*V*_sig_|/*V*_*n*,*rms*_. There is no universal SNR threshold for robust sensing, because the required margin depends on the assay, measurement bandwidth, averaging time, drift, and acceptable detection error. Similarly, there is no universally optimal FET dimension, as device geometry affects both signal coupling and noise. Geometry should therefore be optimized according to the spatial characteristics of the target signal and the associated SNR trade‐off. This trade‐off is particularly important for slowly evolving chemical and biological signals measured in the low‐frequency regime, where 1/*f* noise is most pronounced.

LFN, typically following a 1/*f* trend on the power spectrum and, in nanoscale devices, sometimes as random telegraph signal (RTS) behavior [37], is therefore a central limitation in nanoscale Si FET sensors. Extensive studies have shown that intrinsic LFN in conventional oxide‐gated SiNWFET sensors is often dominated by carrier trapping and detrapping at or near the gate‐dielectric/channel interface [20, 42] rather than with the silicon body alone. This understanding clarified that device scaling can not only enhance electrostatic coupling to surface charges, but also increases the impact of gate‐dielectric/channel traps by reducing statistical averaging. Therefore, sensor optimization must balance signal enhancement with gate‐referred noise reduction, shifting the design focus from sensitivity alone toward SNR and effective resolution. In electrolyte‐gated operation, the situation becomes even more complex because the sensing surface is chemically active. Reversible interfacial processes, including protonation/deprotonation, ion adsorption/desorption, and surface‐state or charge‐transfer kinetics, can generate low‐frequency surface‐potential fluctuations when their time constants fall within the measurement bandwidth. By contrast, slower nonstationary processes, such as ion penetration, hydration, irreversible charge relaxation, and surface reorganization, mainly contribute to drift and baseline instability [21, 43, 44]. Thus, the noise floor and stability of an electrolyte‐gated nanoscale Si FET sensor is a system‐level property of the coupled transistor, sensing interface, electrolyte environment, reference electrode, and readout circuitry.

Figure 1e summarizes the evolution of the area‐normalized gate‐referred voltage noise at 10 Hz, *A*×*S*_vg_, collected from representative reports [17, 20, 21, 22, 23, 24, 29, 30, 31, 32, 33, 34, 35]. To make this comparison transparent, the main benchmarking conditions are summarized in Table 1. The active gate‐coupled area is used in the original report or estimated from the reported device geometry, and *S*_vg_ is obtained from *S*_id_/*g*_m_^2^, in which *S*_id_ is drain‐current power spectrum density and *g*_m_ is gate transconductance, or directly reported as an equivalent input‐voltage noise. When noise values were reported at different frequencies, values at or near 10 Hz were used whenever available; otherwise, conversion was only considered when the reported spectrum followed an approximately 1/*f* dependence. Because the compared devices differ in bias point, electrolyte condition, reference‐electrode configuration, and area definition, Figure 1e should be interpreted as a semi‐quantitative roadmap rather than a strict one‐to‐one performance ranking. The main purpose of the roadmap is to show that, with improvements in semiconductor processing, interface passivation, electrostatic design, and gate architecture, the intrinsic *A*×*S*_vg_ of nanoscale Si FET sensors has been progressively reduced toward reported benchmark levels of solid–liquid interfacial noise measured under defined surface and electrolyte conditions, for example, 1∼3 × 10^−10^ µm^2^V^2^/Hz [21]. Once the device noise approaches this regime, the electrolyte‐facing interface is no longer merely a sensing layer; it becomes an experimentally observable and potentially dominant noise source. Further reduction of internal transducer noise is therefore essential not only for improving sensor resolution, but also for directly resolving interfacial fluctuation mechanisms, including proton binding/debinding, ion adsorption/desorption, surface‐state dynamics, charge‐transfer fluctuations, and impedance‐related electrochemical voltage noise [21, 23, 43].

**TABLE 1:** Benchmarking conditions for the noise roadmap shown in Figure 1e.

Refs.	Device	Gate structure	Test environment	Testing frequency	Active area	*A*×*S*_vg_ at 10 Hz (µm^2^V^2^/Hz)
[17]	SiNW bioFET	APTES/Si	PBS/KCl solution	1 Hz	100 nm × 5 µm	8.5 × 10^−9^
[20]	SiNW‐ISFET	Al_2_O_3_/Si	DI water	10 Hz	1 µm × 6µm	6.0 × 10^−10^
[21]	electrolyte /TiN	TiO_x_/TiN	KCl solution	10 Hz	300 µm diameter	1∼3.0 × 10^−10^
[22]	SJGFET	PtSi/Si	Dry	10 Hz	90 nm × 900 nm	1.3 × 10^−10^
[23]	SJGFET	PtSi/Si	Dry	10 Hz	500 nm × 900 nm	1.3 × 10^−11^
[24]	SiNW‐ISFET	Bare Si	KCl solution	10 Hz	not explicitly reported	1.0 × 10^−10^
[29]	ISFET	Oxide/Si	pH buffer	1 Hz	600 µm × 20 µm	2.4 × 10^−9^
[30]	ISFET‐LBJT	SiO_2_(ALD)/Si	pH buffer	10 Hz	63 µm × 0.96 µm	2.5 × 10^−9^
[31]	SiNW‐ISFET	SiO_2_(Thermal)/Si	PBS	10 Hz	50 nm × 10 µm	5.0 × 10^−9^
[32]	SiNW‐ISFET	SiO_2_(Thermal)/Si	NaCl solution	10 Hz	15 nm × 50 nm	1.5 × 10^−10^
[33]	SiNW‐ISFET	HfO_2_/SiO_2_/Si	pH buffer	1 Hz	5 µm × 0.09 µm	1.1 × 10^−10^
[34]	pMOS‐FinFET	SiON/Si	PBS	1 Hz	10 nm × 130 nm	5 × 10^−11^
[35]	FinFET	SiON/Si	KCl solution	10 Hz	20 nm × 10 µm	8 × 10^−11^

The transition from sensitivity‐centered design to SNR‐centered sensing has been addressed through several complementary routes in the literature. These include improving dielectric/channel interface quality, engineering the current path, reducing sensing‐surface fluctuations, stabilizing the reference/electrolyte environment, and improving the readout chain. Within this broader context, our previous studies provide representative examples at different levels of the signal‐transduction chain. On the sensing interface side, our studies of solid–liquid interfacial noise connected potentiometric low‐frequency fluctuations with the thermodynamics and kinetics of proton binding at amphoteric surface sites [21, 43]. These results indicate that the sensing surface should be regarded as an active, dynamic interface rather than a passive pH‐to‐potential converter. On the device side, we developed Schottky‐junction‐gated silicon nanowire FETs (SJGFETs) [22, 23, 45, 46, 47], in which the noisy gate oxide/channel interface in a conventional metal‐oxide‐semiconductor (MOS) gate structure is replaced by a metal/semiconductor Schottky junction. Because traps at the gate‐oxide/channel interface are a major intrinsic noise source in conventional SiNWFETs, this architecture provides an architectural route to suppress MOS‐interface trap‐related noise by removing the trap‐rich gate‐oxide/channel interface from the main transduction path, rather than relying only on incremental gate dielectric optimization. By further optimizing the bottom buried oxide/Si interface in a tri‐gate device geometry, ultralow area‐normalized gate‐referred noise on the order of 10^−11^ µm^2^V^2^/Hz at 10 Hz was achieved under dry device‐level electrical characterization [23], reaching a level below previously reported solid–liquid interfacial noise. This intrinsic transducer noise is below previously reported benchmark levels of solid–liquid interfacial noise [21], proving that the transistor noise can be reduced to a regime where the electrolyte‐facing interface becomes the dominant noise contribution.

High‐SNR sensing, however, cannot be achieved by intrinsic transducer optimization alone. As the intrinsic device noise floor is reduced, non‐transistor contributions become increasingly important, including drift, reference‐electrode instability, flow‐induced artifacts, parasitic electrical pickup, and external readout noise. For instance, flow‐induced artifacts refer to streaming potentials, pressure‐induced reference‐potential shifts, or transient concentration changes during solution exchange, whereas parasitic pickup includes power‐line interference and capacitive coupling through cables, probe pads, reference electrodes, or high‐impedance liquid‐gate nodes. To preserve the sensing signal in the readout chain, we explored integration of a low‐noise lateral bipolar junction transistor (LBJT) [48, 49, 50, 51, 52] with a SiNRFET on a SOI substrate, aiming to immediately amplify weak signals from the nanoscale sensor before they are degraded by external interferences. More recently, AC‐bias‐stabilized SiNRFET platforms integrated with microscale Ag/AgCl pseudo‐reference electrodes have addressed long‐term electrolyte‐gated operation in microfluidic environments by suppressing ion‐penetration‐induced drift, minimizing streaming‐potential artifacts, and improving baseline stability [53]. Together, these studies support a device‐to‐system perspective: low‐noise field‐effect sensing requires codesign of the transducer, sensing surface, reference electrode, microfluidic environment, and readout circuitry.

In this paper, we review LFN in nanoscale Si FET sensors from this hierarchical perspective. We first summarize the principal physical models used to interpret LFN, including Hooge mobility fluctuation [54], McWhorter carrier‐number fluctuation, correlated carrier‐number and mobility fluctuation [39, 42, 55, 56, 57, 58, 59], and solid–liquid interfacial noise models [21, 43]. We then move to noise‐mitigation and stability‐improvement strategies at different levels of the signal‐transduction chain, following silicon‐gate dielectric interface engineering [60], current path control [41], sensing surface engineering [24, 33], Schottky junction gating [22, 23], bias‐programmed drift stabilization [53], and local amplification [50, 51]. Finally, we highlight high‐SNR sensing applications that particularly benefit from low‐noise nanoscale Si FET transducers, including molecular diagnostics [61, 62], ion and pH sensing [63], cellular and microbial metabolism monitoring [64, 65, 66, 67], rapid antibiotic susceptibility testing [68], and dense chemical imaging [69, 70, 71, 72, 73, 74]. The central message is that future FET biosensors should be evaluated not only by sensitivity, but by SNR, LoD, stability, and the ability to preserve weak sensing signals throughout the signal readout chain.

## 2 Principles and Models of LFN in Nanoscale Si FET Sensors

### 2.1 Introduction to LFN in Nanoscale Si FET Sensing System

LFN in FET‐based sensors is commonly characterized by *S*_id_, the normalized current noise, *S*_id_/*I*_d_^2^, or *S*_vg_. Among these metrics, the gate‐referred representation is particularly useful for sensor evaluation because it converts the measured output‐current fluctuations into the equivalent gate voltage‐referred input noise. For a FET‐based biosensor, the primary sensing event, such as a pH change, ion activity variation, or biomolecular binding, is first expressed as a surface‐potential change of the sensing layer and is subsequently transduced into a drain‐current response. Therefore, a lower *S*_vg_ corresponds directly to a smaller resolvable surface‐potential change, provided that the sensitivity, calibration, and baseline stability are well defined.

Figure 2 [75] summarizes the major noise sources in an electrolyte‐gated FET sensing system. Intrinsic device‐level contributions include carrier number fluctuation and mobility fluctuation, which often originate from carrier trapping and detrapping at or near the dielectric/semiconductor interface, as well as thermal noise and shot noise [20, 38, 42, 76, 77]. During electrolyte‐gated operation, additional noise can arise from the liquid‐facing interface, including stochastic protonation/deprotonation reactions, ion adsorption/desorption, surface‐state dynamics, charge‐transfer processes, and impedance‐related electrochemical voltage noise [21, 43, 44]. We recently discovered that the dynamic interactions at the solid–liquid interface are the dominant contribution to the noise originating from the liquid environment. The observed 1/*f*‐like spectrum can therefore originate from different microscopic processes. When one or a few traps dominate the conductance fluctuation, the time‐domain signal may exhibit RTS behavior [37, 78], characterized by discrete switching between current levels. When many gate dielectric traps with a broad distribution of capture and emission time constants contribute simultaneously, the superposition of their Lorentzian spectra gives rise to a smoother 1/*f*‐like noise spectrum [77, 79]. In addition to the intrinsic device noise and extrinsic liquid noise, the measured spectrum may contain substantial system‐level contributions. These system‐level contributions include reference‐electrode instability, mechanical vibration, illumination‐induced perturbations, temperature fluctuations, cable pickup, leakage through packaging or passivation layers, and external measurement electronics noise [80]. Depending on their time scale and origin, they may appear as broadband noise, discrete spectral peaks, baseline drift, or intermittent time‐domain artifacts rather than as intrinsic 1/*f* noise. Consequently, a measured low‐frequency spectrum cannot be exclusively assigned to the transistor channel based on spectral shape alone. Therefore, reliable identification of the dominant noise source requires a combination of spectral analysis and controlled experiments. Bias dependence can help distinguish carrier‐number fluctuation from mobility‐related effects; geometry scaling can reveal whether the gate‐referred noise follows the expected inverse‐area dependence, *S*_vg_∝1/*A*, as expected for statistically averaged trap or interfacial fluctuations; dry and electrolyte‐gated measurements can separate transistor‐side noise from solid–liquid interfacial noise; and reference‐electrode, light, temperature, and instrumentation controls are necessary to exclude external artifacts. This system‐level view is essential for total noise suppression. Effective reduction of LFN in nanoscale Si sensors requires engineering not only the transistor channel, but also the sensing dielectric, solid–liquid interface, reference electrode, and readout chain, as discussed in the following sections.

**FIGURE 2:**
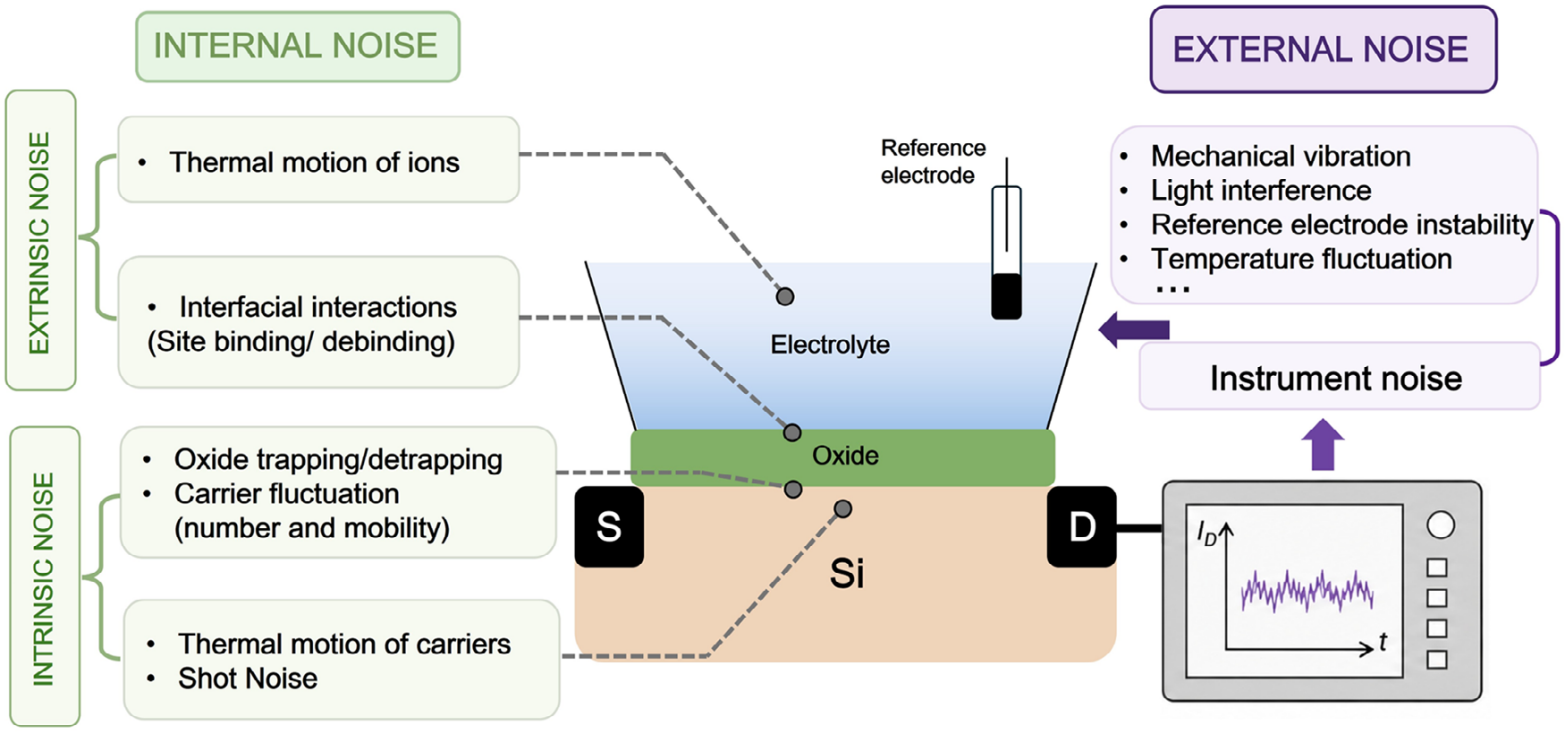
Noise sources appearing in an electrolyte‐gated FET sensing system consisting of device (intrinsic), electrolyte (extrinsic), and characterization instrument (adapted from Ref [75], open access, 2017, DiVA(UU)).

When assessing the noise performance, it is also important to distinguish area‐normalized gate‐referred noise from the absolute gate‐referred noise floor. The metric *A*×*S*_vg_ is useful for comparing the intrinsic quality of different device platforms or interfaces because it partly removes the geometrical area dependence of stochastic fluctuations. However, the practical sensing resolution is determined by the absolute *S*_vg_, or more directly by the integrated RMS gate‐referred noise within the measurement bandwidth. For a given *A*×*S*_vg_, the absolute *S*_vg_ still increases as the active sensing area decreases, indicating that ultralow area‐normalized noise does not automatically guarantee a low absolute noise floor in aggressively scaled sub‐100‐nm devices. More importantly, a single‐frequency value of *S*_vg_ does not directly determine the practical detection resolution. The relevant quantity is the RMS gate‐referred noise integrated over the measurement bandwidth, *V*_n,rms_, which determines the SNR for a given gate‐referred sensing signal. For approximately 1/*f* noise, extending the measurement toward lower frequencies can substantially increase the integrated noise even when the noise spectral density at a reference frequency remains unchanged. Thus, *A*×*S*_vg_ at a defined frequency should be viewed primarily as a technology‐benchmarking metric, whereas the absolute bandwidth‐integrated RMS noise, together with drift and stability, ultimately determines the practical sensing limit. For example, realistic scaling of SJGFETs requires not only low *A*×*S*_vg_, but also uniform Schottky barrier control, low gate leakage, sufficient transconductance, stable passivation, high‐quality remaining interfaces, and suppression of solid–liquid interfacial noise. Thus, *A*×*S*_vg_ should be viewed as a platform‐quality metric, whereas absolute noise, drift, and bandwidth‐integrated resolution ultimately determine the practical sensing limit.

### 2.2 Solid‐State FET Noise Models: Hooge, McWhorter, and CNF+CMF

The simplest phenomenological description of the LFN in FET devices is the Hooge mobility fluctuation model [54] (Figure 2a), in which the normalized current noise scales approximately as *S*_id_/*I*_d_^2^ = *q*α_H_/*fN*. Here, *α*_H_ is the Hooge parameter, and *N* is the number of mobile carriers. The model captures the empirical observation that normalized noise decreases when more carriers participate in conduction. It is useful for quick comparison, but does not explicitly describe carrier exchange with oxide traps, which is often the central process in nanoscale MOS‐type FET sensors.

The McWhorter carrier‐number‐fluctuation model [81] provides a more interface‐specific picture (Figure 2b). Carriers tunnel between the channel and trap states distributed in or near the gate dielectric. Because tunneling time depends exponentially on trap distance from the interface, a spatial distribution of traps produces a broad distribution of capture and emission time constants. The resulting sum of Lorentzian spectra can generate a 1/*f*‐like spectrum. The gate‐referred noise can be described by the following equation:

$$S_{vg}=\frac{kTq^{2}}{8wLC_{ox}^{2}\alpha_{t}}\frac{N_{ot}\left(E_{F}\right)}{f}$$

where *k*T is the thermal voltage, *w*, *L* are the channel width and length, respectively, *C*_ox_ is gate oxide capacitance per unit area, *N*_ot_(*E*_F_) is the oxide trap density near the channel Fermi level, and *α*_t_ is the tunneling attenuation coefficient. This expression captures the characteristic 1/*f* dependence and the inverse scaling with channel area. A key signature is that the current noise is linked to transconductance by *C*_ox_, indicating fluctuations in trap occupancy modulate the effective channel charge and therefore the drain current.

The McWhorter model describes LFN as carrier‐number fluctuation induced by stochastic trapping and detrapping of channel carriers by oxide or interface traps. In this case, the equivalent flat‐band voltage noise [40], *S*_vfb_, is converted into drain‐current noise through the transconductance, giving *S*_id_ = *S*_vfb_ *g*_m_^2^. However, a charged trap can also modify the local Coulomb scattering potential and thereby perturb the carrier mobility, especially in small or ultrathin‐body devices (Figure 3c). Because the carrier number and mobility fluctuations originate from the same trap occupancy fluctuation, the two contributions are correlated. This leads to the carrier number and correlated mobility fluctuation (CNF+CMF) model [39, 42, 55, 56, 58, 59]:

$$S_{id}\left(f\right)=S_{vfb}g_{m}^{2}{\left(1+\Omega \frac{I_{D}}{g_{m}}\right)}^{2}$$

where Ω represents the strength of trap‐induced mobility scattering. When Ω = 0, the expression reduces to the pure carrier‐number fluctuation model. A finite Ω indicates that the same trapped charges responsible for threshold‐voltage fluctuation also contribute to mobility fluctuation through Coulomb scattering. The model is especially relevant for SiNWFETs, SiNRFETs, and thin SOI devices, where a trap located near the interface can have a large effect on the entire current path [82, 83, 84, 85].

**FIGURE 3:**
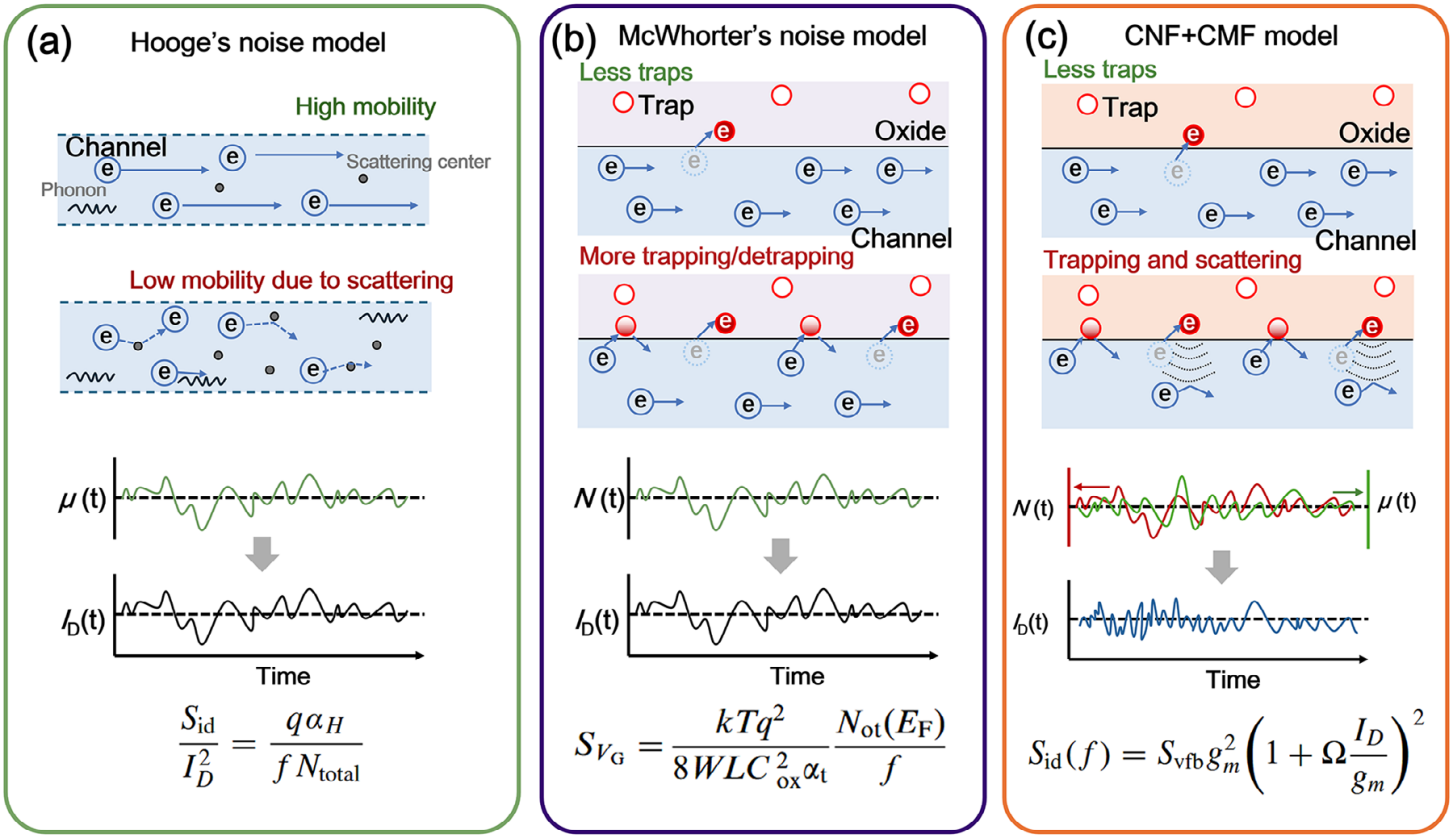
Classical LFN models for FET devices, including schematics of carrier‐trap interaction, current fluctuation, and noise equations. (a) Hooge mobility fluctuation describes noise through fluctuations in carrier mobility and total carrier number. (b) McWhorter carrier‐number fluctuation describes trapping and detrapping between channel and oxide traps. (c) Carrier number fluctuation (CNF) with correlated mobility fluctuation (CMF) model combines number fluctuation with trap‐induced Coulomb scattering, which is particularly relevant in nanoscale and interface‐dominated channels.

Figure 3 illustrates the noise mechanisms and compares these models visually, where Hooge noise emphasizes mobility fluctuation along the channel, McWhorter noise emphasizes trapping and detrapping across the oxide/channel boundary, and CNF+CMF treats trapping and scattering as correlated effects. For nanoscale Si FET sensors, the practical lesson is that model selection should be tied to the physical location of the dominant fluctuation source. If the noise originates mainly in bulk mobility fluctuations, increasing carrier number and improving crystal quality may help [86]. If it originates at a gate dielectric interface, oxide quality [87, 88], passivation, annealing [89, 90], and electrostatic coupling [41] become more important. If it originates at the electrolyte‐sensing layer interface, neither Hooge nor McWhorter alone is sufficient.

A sensor operated in liquid is not just a MOSFET with a different gate terminal, but it is an electrochemical transistor whose gate/liquid boundary is chemically active. The validity of these models therefore depends on where the dominant noise sources are located and how they are coupled to the channel. Hooge‐type mobility fluctuation is most useful as an empirical description when the noise is dominated by mobility fluctuations in the conducting channel and scales inversely with the number of mobile carriers. The McWhorter carrier‐number‐fluctuation model is most appropriate when oxide or near‐interface trap occupancy modulates the channel charge, typically giving a strong relation between *S*_id_ and *g*_m_^2^ and an inverse dependence on gate‐coupled area. The CNF+CMF model becomes important in nanoscale or ultrathin‐body channels, where the same trapped charge that modulates carrier number also perturbs mobility through Coulomb scattering. However, none of these models alone is sufficient when the dominant fluctuation source is located at the electrolyte‐facing interface, in the reference electrode, in the packaging/passivation leakage path, or in the external readout chain. In such cases, dry versus liquid comparison, bias dependence, geometry scaling, electrolyte composition dependence, reference electrode controls, light/temperature controls, and readout chain controls are required to identify the dominant noise source.

### 2.3 Solid–Liquid Interfacial Noise

The noise models introduced above are based on LNF from the solid‐state part of the FET device. However, when a FET sensor operates in a liquid electrolyte, its sensing interface is a chemically active solid‐electrolyte boundary, where reversible surface binding/debinding, ion adsorption/desorption, and charge transfer processes [91, 92] can generate surface‐potential fluctuations when their time constants fall within the measurement bandwidth. In contrast, slower or nonstationary processes, such as irreversible charge relaxation, ion penetration, hydration, and surface reorganization, mainly contribute to drift or baseline instability. Interfacial impedance [93, 94] provides a measurable link between dissipative interfacial processes and voltage noise through the fluctuation‐dissipation relation. These interfacial fluctuations are directly coupled to the channel as equivalent gate‐voltage noise. Therefore, the noise floor of an electrolyte‐gated FET cannot be fully understood without considering the dynamic properties of the sensing solid–liquid interface.

The theoretical basis for describing the solid–liquid interface is the site‐binding model combined with the EDL description [95, 96]. Taking the pH sensing oxide layer as an example, the surface ‐OH groups can release or accept H^+^ ions depending on the pH in the bulk solution, through which the sensing surface forms energy balance with the H^+^ ions and provides surface pH buffer capability. When the bulk pH changes, the corresponding change in the electrochemical potential of H^+^ ions will be shared by the surface buffer capability and the EDL capacitance. Therefore, for an oxide surface with a large number of binding sites and a high surface buffer capacity, most of the change in the bulk H^+^ electrochemical potential is converted into a potential drop across the electrical double layer, resulting in a near‐Nernstian pH response. This model provides a static equilibrium description of the pH‐sensing response, in which the ensemble‐averaged protonation state determines the mean surface potential. However, it does not directly describe the noise spectrum, which arises from temporal fluctuations of the surface protonation state around this equilibrium value. A dynamic description is therefore required to relate stochastic proton binding/debinding kinetics to the measured low‐frequency voltage fluctuations.

A more general description can be obtained from the fluctuation‐dissipation theorem [97]. In this approach, the interfacial voltage noise (*S*_V,int_) is related to the dissipative component of the electrochemical impedance,

SV,intf=4kBTReZintf

where *Z*_int_(*f*) is the impedance of the solid–liquid interface. This expression is important because it connects potentiometric LFN with a measurable electrochemical quantity. Any interfacial process that contributes to the real part of the impedance, such as surface reaction resistance, adsorption/desorption dynamics, charge‐transfer processes, dielectric relaxation, or diffusion‐related dissipation, can generate voltage fluctuations. The impedance formulation therefore shifts the interpretation of solid–liquid interfacial noise from a purely empirical 1/*f* spectrum to a physically measurable interfacial dissipation process. Equivalent‐circuit models [98, 99] are often used to describe *Z*_int_(*f*) in Figure 4a. The EDL capacitance, *C*_DL_, represents non‐Faradaic charge storage associated with ionic redistribution near the solid–liquid interface. Reversible adsorption/desorption and surface‐reaction kinetics are represented phenomenologically by *Z*_ads_; for a pH‐sensitive oxide surface, protonation/deprotonation of amphoteric surface hydroxyl groups is a specific example of such surface‐reaction dynamics. A separate Faradaic branch is represented by the charge‐transfer resistance, *R*_ct_, together with the diffusion‐related Warburg impedance, *Z*_W_. Here, *R*_ct_ describes electron‐transfer kinetics across the solid–liquid interface, whereas *Z*_W_ represents mass‐transport limitations associated with diffusion of ionic or redox species involved in the Faradaic reaction. The series connection of *R*_ct_ and *Z*_W_ therefore represents charge‐transfer kinetics and mass transport acting on the same Faradaic reaction pathway, whereas *Z*_ads_ represents a distinct reversible surface‐reaction pathway that does not necessarily involve electron transfer across the interface. The bulk‐electrolyte impedance, *Z*_bulk_, represents ionic transport through the solution and, in the simplest approximation, is dominated by the solution resistance. These interfacial branches respond to the same small‐signal interfacial potential perturbation and are therefore represented as parallel contributions to the total interfacial admittance. Through the fluctuation‐dissipation relation, the dissipative components of the resulting impedance contribute to equilibrium voltage noise, whereas an ideal capacitance does not itself generate thermal noise but shapes the frequency dependence of the measured fluctuations.

**FIGURE 4:**
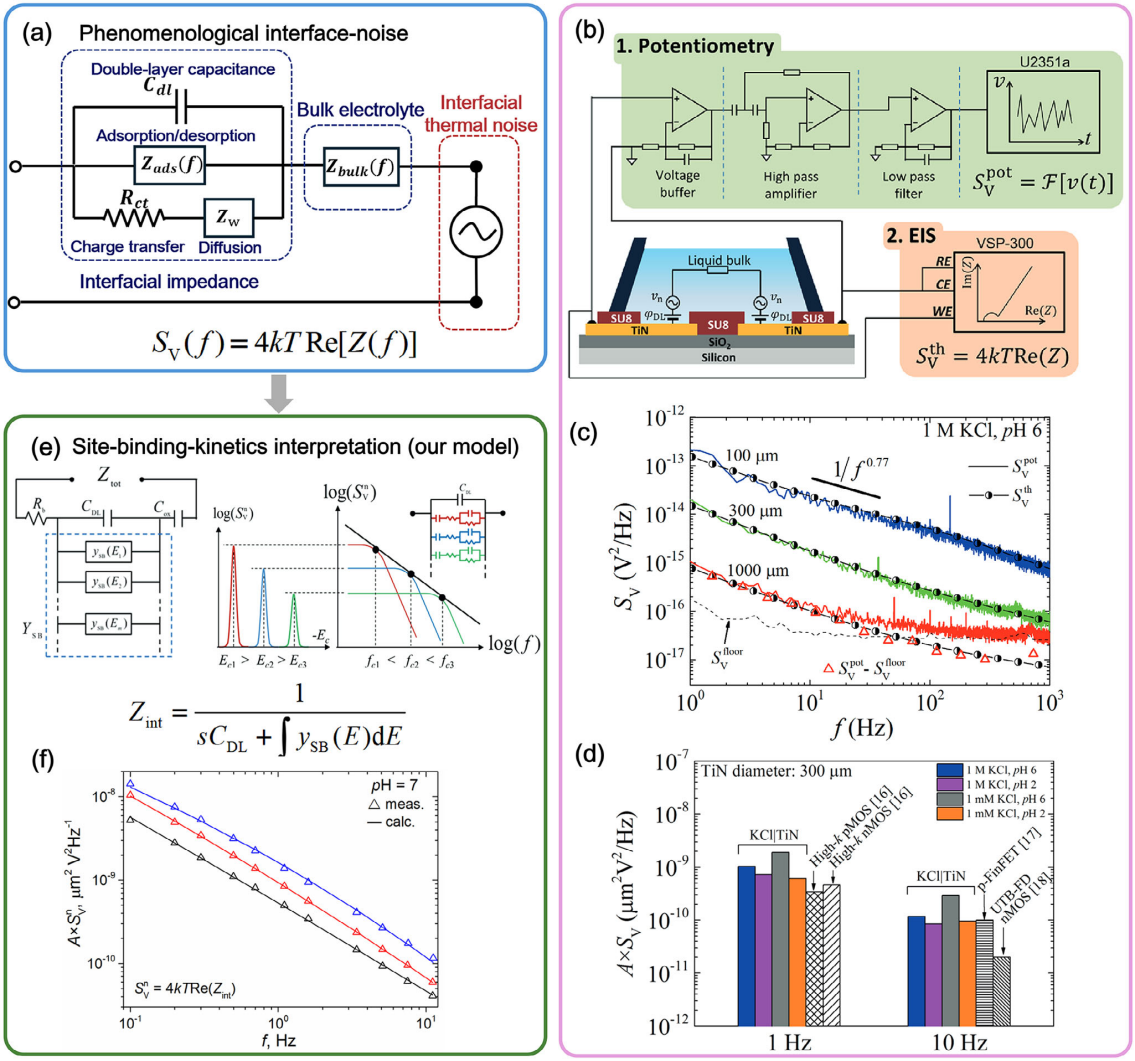
(a) Phenomenological equivalent‐circuit representation of solid–liquid interfacial noise. (b) Experimental setup for validating the impedance‐noise relation by comparing direct potentiometric noise measurements with electrochemical impedance spectroscopy on TiN/electrolyte interfaces [21]. (c) Measured potentiometric noise spectra and impedance‐derived thermal‐noise spectra [21]. (d) Area‐normalized voltage noise benchmark at 1 and 10 Hz [21]. (e) Dynamic site‐binding impedance model [43], including (left) equivalent circuit of the oxide/electrolyte interfacial impedance, with the distributed Y_SB_ in its discrete representation and (right) schematic LFN spectrum contributed from three site‐binding reaction branches with different kinetic energy barriers. (f) Comparison between measured and calculated spectra validates the site‐binding‐kinetics interpretation [43]. (adapted with permission (b‐d) from Ref [21]. Copyright 2016, AIP; (e‐f) from Ref [43]. Copyright 2017, ACS).

In addition to 1/*f*‐like interfacial noise arising from stochastic binding/debinding, ion‐gated FETs may also exhibit thermal and nonequilibrium current‐noise components generated within the electrolyte and electrochemical branches of the ion‐gate network [94, 100]. The frequency‐dependent impedance network consists of the solution resistance (*R*_s_), *C*_DL_, *R*_ct_, interfacial‐state admittance, reference‐electrode impedance, and possible leakage paths through the passivation or sensing dielectric [101, 102]. In a Norton‐equivalent representation, dissipative branches such as *R*_s_, *R*_ct_, and interfacial‐state conductance generate equilibrium thermal current noise, whereas net Faradaic or leakage currents may introduce shot‐like or other nonequilibrium excess noise. Any such current noise (*S*_i_(*f*)) source is converted into equivalent gate‐voltage noise (*S*_v_(*f*)) according to *S_v_* (*f*) = ∣*Z*_eff_(*f*)∣^2^ *S_i_* (*f*), in which *Z*_eff_ is effective impedance. A single lumped RC time constant produces a Lorentzian or low‐pass‐shaped spectrum rather than true 1/*f* noise. However, an approximately 1/*f*‐dependent spectrum can emerge when the ion‐gate impedance contains a broad distribution of relaxation times associated with heterogeneous binding kinetics, distributed interfacial states, or diffusion processes. Therefore, even a white current noise source can produce a frequency‐shaped voltage‐noise spectrum after passing through the *R*_s_, *C*_DL_, *R*_ct_, and interfacial‐state network. Distinguishing this contribution from transistor‐side noise requires electrochemical impedance spectroscopy (EIS), bias‐window dependence, electrolyte‐composition dependence, dummy‐electrode controls, reference‐electrode controls, and dry‐versus‐liquid comparisons.

In our previous works, we first directly characterized the solid–liquid interface noise using two identical TiN electrodes of various sizes for differential measurement in KCl‐based electrolytes without recourse to a reference electrode [21], as illustrated in Figure 4b. The directly measured low‐frequency voltage noise agreed well with the thermal‐noise spectrum independently calculated from the measured interfacial impedance (Figure 4c), supporting its thermal origin. Furthermore, the noise spectral density decreased with increasing electrode area, while the area‐normalized noise converged to a similar range (Figure 4d), supporting the conclusion that the dominant measured contribution originated from the solid–liquid interface. Importantly, this TiN/electrolyte experiment established the interfacial and thermal nature of the measured noise but did not uniquely identify protonation/deprotonation as its sole microscopic origin. It should therefore be regarded as a phenomenological benchmark of solid–liquid interfacial noise rather than as a direct microscopic measurement of proton‐binding kinetics. The TiN/electrolyte system is not intended to quantitatively represent all dielectric‐electrolyte sensing interfaces, since oxide surfaces such as SiO_2_, HfO_2_, Al_2_O_3_, and Ta_2_O_5_ have different surface‐site densities, binding kinetics, charge‐transfer properties, ion‐penetration behavior, and interfacial admittances. Rather, this experiment provides a direct benchmark demonstrating that solid–liquid interfaces can generate measurable impedance‐governed voltage noise, while dielectric/electrolyte interfaces require material‐specific models such as the dynamic site‐binding model discussed below.

To establish a microscopic connection between interfacial noise and proton‐binding dynamics, we subsequently applied a dynamic site‐binding model to ultrathin TiO_2_ sensing layers deposited on TiN electrodes [43]. In this framework, protonation/deprotonation occurs at amphoteric sites on the TiO_2_/electrolyte interface. The corresponding surface‐reaction kinetics are represented by a distributed site‐binding admittance, *Y*_SB_, which is placed in parallel with the *C*_DL_, to form the interfacial impedance *Z*_int_ (Figure 4e). Individual reaction branches are associated with different kinetic barriers and characteristic relaxation times. The superposition of a broad distribution of these relaxation processes produces the observed low‐frequency spectrum. The voltage‐noise spectrum calculated from the resulting interfacial impedance through the fluctuation‐dissipation relation agrees well with the experimental spectrum (Figure 4f), supporting the site‐binding‐kinetics interpretation. This model links solid–liquid interfacial noise directly to surface reaction kinetics and suggests that a low‐noise sensing surface should minimize slow, kinetically heterogeneous proton‐binding sites, rather than simply maximizing pH sensitivity or total site density. Taken together, Figure 4c–f illustrates the progression from phenomenological observation to microscopic interpretation. Figure 4c experimentally validates the relation between dissipative interfacial impedance and thermal voltage noise, while Figure 4d uses electrode‐area scaling to further support the interfacial origin of the measured fluctuations. Figure 4e then introduces a microscopic description in which distributed proton‐binding kinetics determine the interfacial admittance, and Figure 4f shows that this model reproduces the measured LFN spectrum.

These considerations provide interface‐level design implications for low‐noise solid–liquid sensing. The sensing surface should minimize slow and kinetically heterogeneous binding sites, because a broad distribution of activation barriers can generate long‐time‐constant potential fluctuations within the measurement bandwidth. The dissipative component of the interfacial admittance should also be reduced by suppressing charge‐transfer leakage, unstable redox reactions, poorly defined interfacial states, and contamination‐mediated adsorption/desorption pathways. Thus, high chemical sensitivity or high surface‐site density alone is not sufficient if the added sites introduce slow or unstable dynamics. Combined EIS and noise spectroscopy are therefore important for evaluating low‐noise interfaces, because the real component of the interfacial impedance directly reflects dissipative fluctuation channels. Specific material, biasing, and system‐level strategies are discussed in the following sections.

## 3 Strategies for LFN Reduction in Electrolyte‐Gated Nanoscale FET Sensors

LFN reduction in nanoscale sensors cannot be achieved by a single universal strategy. The measured noise floor is determined by the complete signal generation, transduction, and readout chain, including the dielectric sensing surface, dielectric/semiconductor channel interface, the semiconductor channel, reference electrode, microfluidic environment, and readout circuitry. Therefore, noise mitigation strategies can be organized hierarchically according to where the dominant noise source is situated and how strongly it is coupled to the channel current. As summarized in Figure 5, the LFN suppression strategies cover modifying dielectric/channel quality [60], modifying current path [41], smoothing channel surface [24], and removing dielectric/channel interfacial traps by using a Schottky junction gate [22, 23]. Moreover, the engineering of sensing surface is particularly important in BioFET applications because the effective signal and stability can be limited by Debye screening, redox‐mediated potential buffering at functionalized metal surfaces, nonspecific adsorption or contamination, and instability or reorganization of the surface functional layer [103, 104, 105, 106], and so forth. Signal drift is also discussed as a practical stability issue, including our recent work on AC gate‐pulse biasing to suppress drift in electrolyte‐gated SiNRFETs [53]. Importantly, these strategies do not reduce all LFN with the same physical mechanism. Some reduce the intrinsic fluctuation source, some weaken the coupling between the noise source and the channel, and others improve the effective SNR by suppressing drift or preserving the original signal.

**FIGURE 5:**
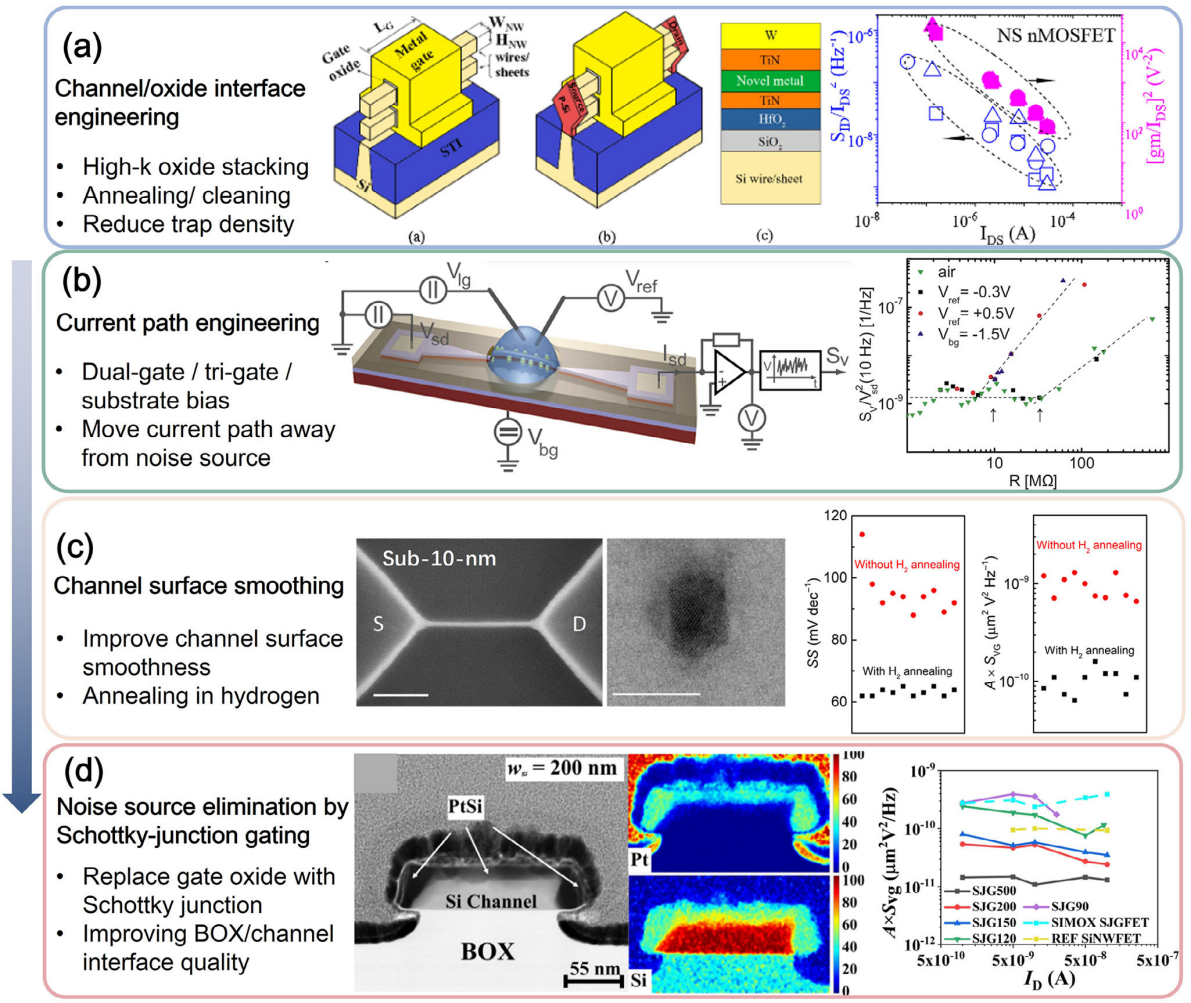
Hierarchical strategies for reducing LFN in nanoscale Si FET sensors. (a) dielectric/channel interface engineering (left) and normalized power spectral density and transconductance over drain current ratio squared as a function of I_DS_ (right) in nanosheet FET [60]. (b) Schematic of dual‐gate FET(left) with voltage noise divided by V_sd_^2^ as a function of resistance at 10 Hz(right) [41]. (c) SEM image of sub‐10‐nm SiNWFET with channel surface smoothed by hydrogen annealing(left) and summary of SS and A × S_VG_ at 10 Hz with and without H2 annealing(right) [24]. (d) XTEM image of the PtSi‐gated SiNW channel together with Pt and Si elemental profiles analyzed by EDX (left) and A × S_vg_ (10 Hz) for SOITEC‐SJGFETs. Best‐achieved A × S_vg_ on the SIMOX‐SJGFETs and the reference SiNWFETs are also included for comparison(right) [23]. (adapted with permission (a) from Ref [60]. Copyright 2020, IEEE; (b) from Ref [41]. Copyright 2011, AIP; (c) from Ref [24] under the terms of the CC BY‐NC license; (d) from Ref [23]. Copyright 2021, IEEE).

To provide a balanced comparison of different low‐noise strategies, Table 2 summarizes representative FET‐based sensor architectures and their main advantages and practical limitations. Conventional MOS‐type ISFETs and SiNWFETs benefit from mature CMOS‐compatible fabrication, scalable integration, and relatively straightforward biasing and calibration, but their noise floor is often limited by dielectric/channel interface traps, oxide‐related drift, and sensing‐surface instability. Current‐path engineering and surface‐smoothing strategies can reduce the impact of these noise sources while retaining the conventional device platform. In contrast, SJGFETs and local bipolar amplification provide more architectural approaches to suppress dominant MOS‐interface noise or preserve weak signals before external readout contamination. These approaches can offer substantial SNR improvement, but they also introduce additional considerations in junction uniformity, leakage control, pixel‐level calibration, process complexity, and long‐term stability. More detailed discussions are provided introduced in the following sections.

**TABLE 2:** Comparison of representative device‐level and readout‐level strategies for reducing noise in nanoscale FET sensors.

Architecture	Noise‐reduction principle	Main advantages	Practical considerations	
High‐quality gate stack / passivation / annealing [60, 107, 108, 109, 110, 111, 112]	Improves dielectric/channel interface and sensing membrane quality	CMOS‐compatible; scalable; mature process flow; compatible with large arrays		Does not remove the oxide/channel interface; residual traps and sensing‐interface noise remain
Dual‐gate / tri‐gate / substrate‐bias engineering [41, 45, 46, 113, 114, 115, 116]	Moves current path away from noisy interfaces or optimizes electrostatic coupling	Improves effective SNR without changing the basic gate stack		Requires careful bias optimization; noise source remains present
Channel surface smoothing [24, 33, 117]	Reduces sidewall roughness, process‐induced defects, and trap density	Improves SNR, hysteresis, and device uniformity		Requires additional process development
SJGFET [22, 23, 45, 46, 47]	Replaces the conventional MOS gate‐control interface with a Schottky‐junction‐controlled channel	Eliminates of gate‐oxide/channel trap‐related noise		Requires substantial process development for uniform Schottky barrier formation, leakage control, passivation, and statistical validation
LBJT / local amplification [48, 49, 50, 51, 52, 118]	Amplifies weak sensor signals locally to suppress interferences from downstream readout noise	Improves overall SNR, especially when the readout/environmental noise is significant		Requires codesign of gain, power, dynamic range, footprint, and calibration

### 3.1 Dielectric/Channel Interface Quality Engineering

In conventional MOS‐type FETs, a major source of LFN is charge trapping and detrapping in the gate dielectric or near the dielectric/channel interface. These traps induce carrier‐number fluctuation and, in many cases, correlated mobility fluctuation through Coulomb scattering. Therefore, improving the dielectric/channel interface in the solid‐state device is the most direct and widely used incremental strategy for reducing intrinsic transistor noise. This approach includes high‐quality gate‐dielectric growth, optimized high‐*k*/interfacial‐layer stacks [107, 108, 109, 110, 111, 112], post‐deposition treatment [119], hydrogen annealing [120, 121, 122], and process cleaning [123] to reduce the trap density. In scaled gate‐all‐around and nanosheet FETs [60] (Figure 5a), the normalized drain‐current noise, *S*_id_/*I*_d_^2^, follows the same bias dependence as *g*_m_^2^/*I*_d_^2^, indicating that the measured flicker noise is well described by the CNF model. The dominant noise is therefore attributed to carrier trapping and detrapping by border traps in the gate dielectric, while the extracted flat‐band referred noise, *S*_Vfb_, serves as a useful figure of merit for evaluating the oxide trap density. It also shows that Coulomb scattering affects mobility, linking oxide‐trap quality to both noise and transport degradation.

For ISFET sensors, this strategy is necessary but not sufficient. A cleaner oxide/Si interface reduces the intrinsic transistor noise, thereby lowering the gate‐referred noise floor. However, the sensing solid–liquid interface remains chemically active and may introduce additional low‐frequency potential fluctuations. Therefore, solid‐interface quality engineering should be regarded as the first level of noise mitigation: it improves the conventional device stack but does not fundamentally remove the gate dielectric‐electrolyte interface from the signal transduction chain.

### 3.2 Electrostatic and Current Path Engineering

A second strategy is to reduce the impact of the existing noise sources on the channel current, instead of eliminating them. This can be achieved by electrostatically controlling the carrier distribution and current path in the FET channel. In dual‐gate, tri‐gate, back‐gate, or substrate‐biased nanowire/nanoribbon FETs [41, 45, 46, 113, 114, 115, 116], the current centroid can be shifted away from a particularly noisy interface, or the operating point can be adjusted to maximize the transduction efficiency relative to the noise.

The importance of electrostatic control is clearly illustrated by dual‐gated SiNRFET sensors [41] (Figure 5b). Tarasov et al. showed that the apparent SNR depends strongly on the operating regime of dual‐gated SiNRFETs. A contact‐dominated regime means that the total resistance is mainly determined by the contact/access regions, which are weakly coupled to the liquid gate, whereas a nanoribbon‐dominated regime means that the exposed nanoribbon channel dominates the resistance and is efficiently modulated by the liquid gate. When the nanoribbon resistance dominates, and the liquid‐gate coupling is strong, the threshold‐voltage noise can be much smaller than in the contact‐dominated regime, leading to an SNR improvement approaching two orders of magnitude. Moving the current path away from a low‐quality interface can reduce the drain‐current noise by weakening the coupling between channel carriers and interface traps. However, the sensor‐relevant metric is the equivalent threshold‐voltage or gate‐referred voltage noise, which depends on both the drain‐current noise and the transconductance/gate coupling. Therefore, current‐path engineering should be evaluated using gate‐referred noise rather than drain‐current noise alone. In the context of Figure 5b, dual‐gate or tri‐gate operation should therefore be described as current‐path engineering rather than true noise‐source suppression. The traps remain present, but their electrostatic influence on the conducting channel is reduced. Electrostatic engineering can substantially improve SNR, especially in nanoscale devices, but the residual noise source still limits further scaling unless the underlying interface quality is also improved.

### 3.3 Channel Surface Smoothing and Defect Passivation

For nanoscale FET sensors, channel‐surface quality directly determines LFN, hysteresis, device‐to‐device variation, and SNR. As the nanowire width decreases, etched sidewalls occupy a larger fraction of the conducting channel, and each surface defect has a stronger electrostatic influence. Line‐edge roughness, ion etching induced damage, dangling bonds, non‐uniform oxidation, and gate‐dielectric thickness variation can therefore enhance carrier‐number fluctuation, Coulomb‐scattering fluctuation, and threshold‐voltage dispersion.

Zafar et al. showed that fabrication‐induced defects near the Si/gate‐dielectric interface and nonuniform channel coverage are major origins of sensor variation [33, 117]. By optimizing resist thickness, electron‐beam dose, dry etching, sacrificial oxidation, and forming‐gas annealing, they obtained smoother nanowire sidewalls and a high‐quality channel/dielectric interface. The optimized 30‐nm SiNWFETs exhibited a near‐ideal subthreshold swing, substantially reduced hysteresis and drift, a 10‐fold improvement in peak SNR, and a low extracted oxide‐trap density. These improvements are consistent with the suppression of stochastic trap‐related noise and slower instability processes by surface smoothing. We further demonstrated the importance of surface reassembly in sub‐10‐nm electrolyte‐gated SiNWFETs [24] (Figure 5c). In such extremely scaled devices, process‐induced roughness becomes comparable to the nanowire diameter and can seriously impact the SNR. We utilized a H_2_ annealing process [24, 120, 121, 122], which promotes surface diffusion and atomic rearrangement of Si atoms on etched sidewalls, thereby producing an atomically smooth channel surface and reducing the area‐normalized gate‐referred noise by about one order of magnitude [24]. This noise reduction was essential for resolving discrete single hydrogen ion binding/debinding events on the nanowire surface. Thus, channel‐surface smoothing is an essential process‐level noise‐reduction strategy. It can also improve surface uniformity and array‐level reproducibility.

### 3.4 Schottky‐Junction Gating: Removing the Noisy Gate‐Dielectric/Channel Interface

A more architectural strategy for reducing intrinsic FET noise is to suppress the dominant trap‐related contribution associated with the conventional MOS gate interface. In MOS‐type SiNWFETs, the gate‐dielectric/channel interface is a major source of LFN because carrier trapping and detrapping near this interface can directly modulate the channel charge and mobility. SJGFETs address this limitation by replacing the conventional MOS gate‐dielectric/channel interface with a PtSi/Si Schottky junction [22, 23]. The device therefore operates in a JFET‐like mode, where the channel conductance is modulated by depletion from the Schottky junction. By removing the noisy gate‐oxide/channel interface from the main gate‐control path, this architecture strongly suppresses MOS‐interface trap‐related noise, rather than relying only on incremental gate‐dielectric optimization.

The first SJGFET demonstration showed that replacing the dielectric/Si channel interface with a PtSi/Si Schottky junction substantially reduced intrinsic device noise compared with both inversion‐mode and depletion‐mode SiNW MOSFET references [22]. The device exhibited near‐ideal subthreshold behavior and an area‐normalized gate‐referred voltage noise of 1.3 × 10^−10^ µm^2^V^2^/Hz at 10 Hz. The next improvement was the tri‐gate SJGFET, in which the PtSi/Si Schottky junction wraps the top and two sidewalls of the SiNW. This geometry improves electrostatic control and allows the current path to be confined away from the buried oxide/Si interface [45, 46]. However, after the conventional gate‐oxide/channel interface is removed from the main gate control path, the bottom BOX(buried oxide)/Si interface can become a dominating noise source, particularly in SIMOX(separation by implantation of oxygen) substrates with poor BOX/Si interface quality. Applying a negative substrate bias or using narrow tri‐gate geometry to push the conduction path away from the BOX/Si interface can significantly improve the LFN performance.

A major LFN performance advance was achieved by transferring the tri‐gate SJGFET concept from SIMOX substrates to bonded SOI substrates (SOITEC) [23] (Figure 5d) with a higher BOX/Si interface quality. The SOITEC‐SJGFET showed near‐ideal gate coupling with a subthreshold swing of approximately 66 mV/dec. More importantly, the area‐normalized gate‐referred noise reached 1.2×10^−10^ µm^2^V^2^/Hz at 1 Hz and 1.1×10^−11^ µm^2^V^2^/Hz at 10 Hz [23], lower than previously reported FET‐based sensor noise and even below reported oxide/electrolyte interfacial noise levels. This result is important for ISFET sensing because it implies that the intrinsic transducer noise can be reduced below the solid–liquid interface noise floor, allowing the sensing interface itself to become the limiting and experimentally observable fluctuation source. Further sensing surface engineering will be discussed in a later section.

The noise mechanism was further refined by a detailed CNF+CMF analysis of SOITEC‐SJGFETs [47]. Unlike SIMOX‐SJGFETs, where Coulomb‐scattering‐related CMF near the BOX/Si interface was significant and could be strongly suppressed by moving the current path away from the interface, SOITEC‐SJGFETs were found to be dominated mainly by carrier‐number fluctuation. The CMF contribution associated with Coulomb scattering near the BOX/Si interface was relatively weak because the bonded SOI interface had much lower trap density. The observed substrate‐bias and width dependence was therefore attributed primarily to the nonuniform energy distribution of BOX/Si interface traps, rather than simply to the distance between the current centroid and the interface. The extracted trap density in SOITEC‐SJGFETs was around three orders of magnitude lower than that in SIMOX‐SJGFETs. To further suppress LFN, the next step is not only better bias optimization, but architectural removal of the remaining BOX/Si interface, for example, by a true gate‐all‐around Schottky junction that wraps all surfaces of the SiNW channel. In this sense, SJGFETs provide a clear device‐level route from interface‐noise mitigation toward intrinsic trap‐related device noise suppression.

Despite the ultralow intrinsic noise demonstrated by SJGFETs, their broader implementation requires further progress in process uniformity, interface passivation, and statistical validation. Uniform formation of metal/semiconductor Schottky junctions around nanoscale channels depends on precise control of the silicide phase, junction geometry, barrier height, and residual interface states, since variations in these factors can affect control voltage, leakage current, transconductance, and noise characteristics. Moreover, although the conventional gate‐dielectric/channel interface is removed from the main gate‐control path, other interfaces, including the metal/semiconductor junction, BOX/Si interface, and electrolyte‐facing passivation layer, may become relevant fluctuation or stability‐limiting elements. For electrolyte‐gated sensing, robust junction passivation, low gate leakage, suppression of electrochemical side reactions, and stable repeated‐bias operation are therefore essential. At the current stage, SJGFETs should be regarded primarily as a laboratory proof‐of‐concept platform demonstrating the low‐noise potential of Schottky‐junction‐based gate control, while further work is needed to establish wafer‐level reproducibility and array‐level integration. Future studies should systematically evaluate device‐to‐device distributions of gate‐referred noise, area‐normalized noise, subthreshold swing, transconductance, Schottky leakage or rectification behavior, control‐voltage variability, hysteresis, and long‐term drift under realistic sensing conditions. Such statistical validation will be critical for determining whether the ultralow‐noise advantage demonstrated in individual devices can be translated into reproducible large‐scale sensing applications.

A similar approach is Bipolar junction transistors (BJT)‐based sensing. BJTs have the lowest voltage‐referred noise since the external potential is transferred directly to the internal base without any intervening layer. Zafar et al. [124, 125] attached a sensing wire to a BJT base, achieving 20× superior voltage‐referred noise compared to ISFETs. However, one must be careful when comparing BJTs to FETs, since the BJT base current can introduce extra noise and drift due to redox reactions needed to support the current. This would be manifested for small sensing areas, as contrasted to the large areas used in that study.

### 3.5 Sensing Surface Engineering for Biomolecular Field‐Effect Sensing

For biomolecular FET sensors, the sensing surface is not only a biochemical recognition layer but also a decisive part of signal generation. Even with a low‐noise SiNWFET or ISFET transducer, the final response can be strongly limited by receptor density, molecular conformation, Debye screening, ionic strength, surface buffering, and molecular‐layer stability.

DNA‐FET sensing [103, 104, 105, 106] illustrates these constraints clearly (Figure 6a–f). In principle, target‐probe DNA hybridization introduces additional negative charge near the gate surface and shifts the FET threshold voltage. However, only charges located within the Debye length can be effectively registered. Under physiological ionic strength, the Debye length is below 1 nm, so most DNA charges are screened. Lowering salt concentration extends the Debye length (Figure 6b), but it also weakens surface hybridization because electrostatic repulsion between DNA strands is stronger (Figure 6c). Therefore, potentiometric DNA sensing faces an intrinsic trade‐off: high salt improves hybridization but suppresses charge registration, whereas low salt improves electrostatic coupling but reduces hybridization efficiency. This adverse effect of low salt concentration on the hybridization efficiency was also found to be more significant on the surface with higher probe coverage due to steric hindrance (Figure 6d). Our Surface plasmon resonance (SPR) analysis of surface DNA hybridization suggests that achieving high‐efficiency hybridization in a high‐salt‐concentration buffer, followed by potentiometric measurements in a low‐salt‐concentration buffer, may yield the optimal potentiometric signal.

**FIGURE 6:**
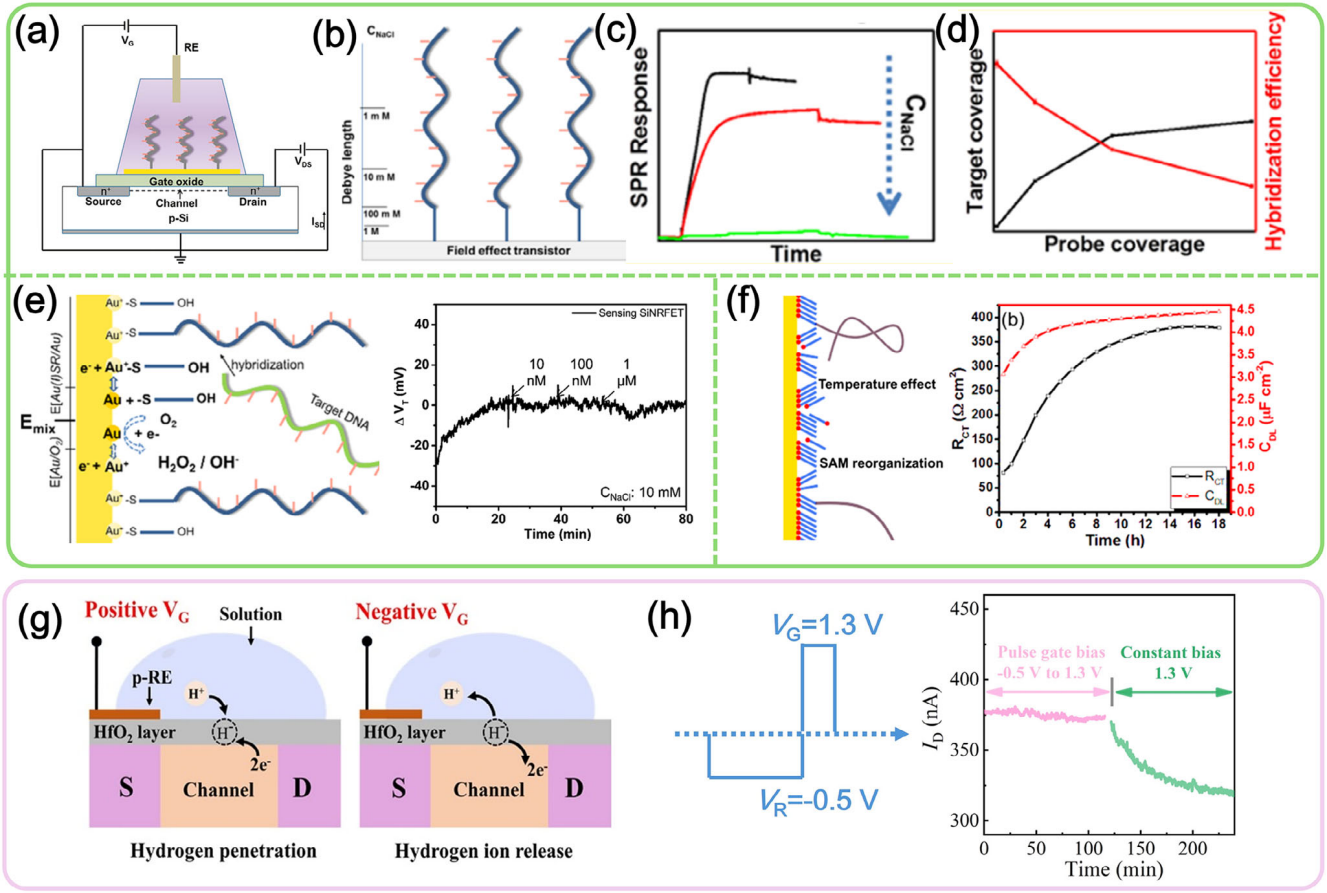
Schematic views of (a) an ISFET DNA sensor and (b) Debye length variation at different NaCl concentrations. (c) SPR response decreases with lower NaCl concentration due to reduced hybridization efficiency. (d) Target DNA coverage increases with probe coverage, but the hybridization efficiency decreases due to steric hindrance. (e) A schematic of redox buffering effect at gold electrode surface(left) and real‐time potentiometric detection of DNA in Tris buffer using sensing SiNRFET, showing screened DNA response (right). (f) A schematic of the aptamer/mercaptohexanol(MCH) reassembling during electrode preparation and hot binding hybridization process(left), and tested charge transfer resistance(R_CT_) and double layer capacitance (C_DL_) drifting(right). (g) A schematic of hydrogen ion migration in the HfO_2_ sensing layer under a positive/negative gate bias. (h) I_D_ drift of a SiNRFET first under AC pulse gate bias, then switches to constant gate bias, showing suppressed ion‐penetration‐induced ISFET drift. (adapted with permission (a‐d) from Ref [106]. Copyright 2020, ACS; (e) from Ref [103]. Copyright 2021, ACS; (f) from Ref [105]. Copyright 2019, ACS; (g‐h) from Ref [53]. Copyright 2025, Elsevier).

However, the expected DNA hybridization signal can be buffered by the redox buffer effect on the sensing surface. In our study of FET‐based potentiometric DNA sensing using a silicon nanoribbon FET with a gold‐sensing gate modified with thiol‐gold chemistry [103], no clear potentiometric signal associated with DNA hybridization was observed, although surface DNA hybridization was confirmed and even quantified by the SPR analysis. This behavior was attributed to a mixed‐potential effect at the thiol‐modified gold electrode. The open‐circuit potential of the electrode is determined by the balance of multiple interfacial redox processes, including Au‐thiol‐related reactions and dissolved‐oxygen‐related redox reactions. These redox processes can pin or buffer the electrode potential, so that the potential change expected from biomolecular binding or DNA hybridization is partially compensated. (Figure 6e). This redox‐buffering effect compensated the charge introduced by DNA hybridization and suppressed the potentiometric signal. Analogous redox‐buffer effects may also occur in other types of potentiometric biomarker detection and therefore must be taken into serious consideration.

Interface stability is equally important. Aptamer/6‐Mercapto‐1‐hexanol(MCH)‐modified gold electrodes showed significant charge transfer resistance(*R*_CT_)and double layer capacitance(*C*_DL_) drift due to MCH self‐assembled‐monolayer reorganization, and hot hybridization buffer induced additional drift [104, 105] (Figure 6f). Gold‐electrode pretreatment and electrochemical preparation conditions, such as surface cleaning, O_2_ plasma/ethanol treatment, CV cycling, reference‐electrode leakage, counter‐electrode contamination, and thiol‐based functionalization, can alter the surface cleanliness, interfacial redox activity, and reproducibility of gold‐based BioFET sensing surfaces [104]. Therefore, biomolecular FET sensing should be treated as an interface‐limited process. High‐SNR detection of biomolecules requires not only low transducer noise, but also a stable, specific, weakly buffered, and electrostatically well‐coupled sensing surface.

### 3.6 Drift Suppression in Nanoscale FET Ion Sensors

In practical FET‐based ion sensing, long‐term drift can become as limiting as stochastic LFN, especially when the target signal evolves slowly, such as in metabolism monitoring [66] or long‐duration biochemical assays. Drift is commonly associated with nonstationary processes at the sensing stack, including hydration, slow charge relaxation, reference‐electrode instability, and ion penetration into the sensing dielectric [44, 53, 126]. For our HfO_2_‐gated SiNRFETs, a particularly important mechanism is hydrogen‐ion penetration under positive gate bias [53] (Figure 6g). Under a positive gate bias, H^+^ or hydrogen‐related species can be driven into the HfO_2_ sensing layer and migrate toward the HfO_2_/Si interface, where they may undergo charge‐state conversion and form negatively charged H‐related defects or trapping centers [127, 128]. The accumulation of negative charge in the gate dielectric then leads to a positive threshold‐voltage drift. This mechanism is supported by the observation that stronger gate bias produces larger Δ*V*_th_ drift and that similar drift is obtained with both on‐chip and commercial reference electrodes, indicating that the drift originates mainly from the SiNRFET rather than the reference electrode.

We proposed an AC pulse gate‐bias strategy to suppress this ion‐penetration‐induced drift by alternating a positive sensing pulse and a negative recovery pulse [53] (Figure 6h). The positive pulse enables normal ISFET readout, whereas the negative pulse promotes oxidation/release of hydrogen‐related species from HfO_2_ back into the electrolyte. Using 1.3 V sensing pulses and ‐0.5 V recovery pulses reduced Δ*V*_th_ drift from about 10 mV/h under constant bias to about 1 mV/h under AC pulse operation; further increasing the negative pulse gave little additional benefit and may risk AgCl degradation.

Other drift‐reduction principles include lowering the DC gate field, reducing sensing duty cycle [129], using denser ion‐blocking dielectrics [130], improving dielectric/passivation quality [131], stabilizing or integrating reference electrodes [132], minimizing streaming potential by placing the reference electrode close to the sensor [133], and applying differential or model‐based drift correction [134]. In summary, complementary methods include material engineering to suppress ion transport, bias programming to reverse accumulated ionic charge, and system integration to remove external baseline artifacts.

## 4 Readout‐Level Noise Suppression and Local Low‐Noise Amplification

A low‐noise transducer is not sufficient if the signal is degraded after leaving the sensing device. The weak output of a nanoscale FET sensor can be contaminated by parasitic capacitance, leakage, cable pickup, reference instability, input‐referred amplifier noise, offset drift, and low‐frequency environmental interference. As the internal noise (including both the solid and liquid noise) is reduced, these external contributions become increasingly visible. Readout design should therefore be considered part of the noise strategy, not an afterthought.

Figure 7 compares three readout‐level strategies. Differential readout, including ISFET/Reference FET pairs and Wheatstone‐bridge‐like structures [135] (Figure 7a), suppresses common‐mode drift, temperature shifts, and environmental interference. Array‐level correction uses source followers [135] (Figure 7b), correlated double sampling [16, 71, 136, 137] (Figure 7c), chopping, auto‐calibration, and fixed‐pattern‐noise reduction to manage pixel variation and circuit offsets. However, these methods mainly correct or filter signals after transduction; they do not prevent a weak sensing signal from being contaminated before the first amplification stage.

**FIGURE 7:**
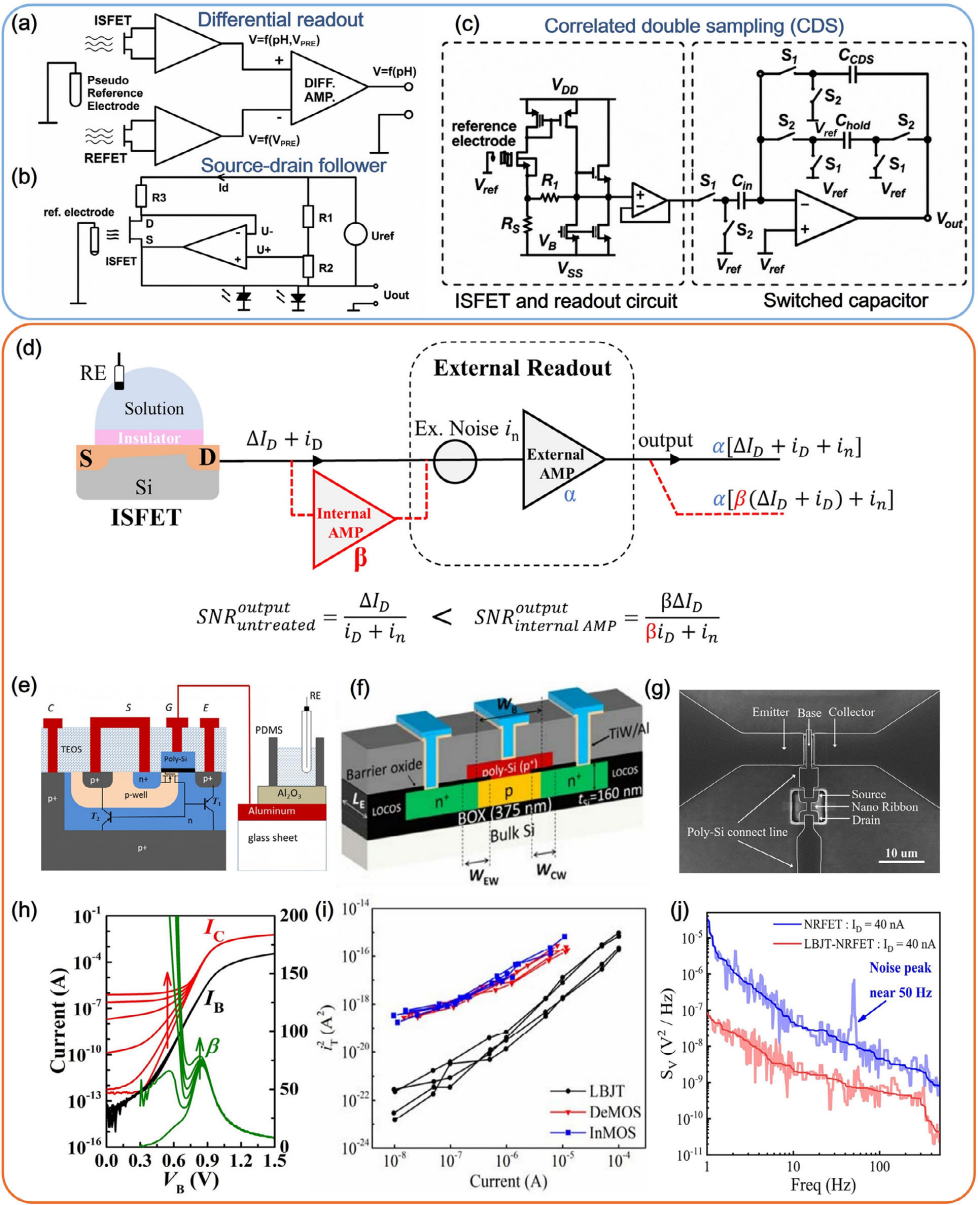
Readout‐level noise suppression and local amplification. (a) Differential readout [135], (b) source‐drain follower [135], and (c) array‐level correlated double sampling (CDS) [136]. (d) Schematic diagram of an ISFET measurement system with an external readout circuit. The red dashed line indicates the signal path when internal amplification is implemented [138]. Our previous work on vertical IGBA [48], LBJT [51] and LBJT‐NRFET [53] shows a local low‐noise amplification approach to suppress external interferences: schematic device structures of (e) the vertical IGBA and (f) the LBJT, (g) top‐view SEM image of a LBJT‐NRFET, (h) Gummel characteristics and current gain of the LBJT [49], (i) current noise comparison between a LBJT and a reference MOSFET [51], and (j) voltage noise spectrum comparison under 50‐Hz noise interference with and without the integration LBJT amplifier [53]. (Figures are adapted with permission (a‐b) from Ref [135]. Copyright 2003, Elsevier; (c) from Ref [136]. Copyright 2007, IET; (d) from Ref [138], open access DiVA(UU); (e) from Ref [48]. Copyright 2014, AIP; (f, i) from Ref [51]. Copyright 2018, IEEE; (g, j) from Ref [53]. Copyright 2024, IEEE; (h)from Ref [49]. Copyright 2020, IEEE).

Local low‐noise amplification addresses this limitation by increasing the signal amplitude immediately at the sensor output. The basic principle (Figure 7d) [138] is that an internal amplifier with gain *β* amplifies both the sensor signal Δ*I*_D_ and intrinsic sensor noise *i*_D_, but not the downstream external noise *c*. Without local amplification, the output SNR is approximately, *SNR*_out_ = Δ*I*_D_/(*i*_D_+*i*_n_). With internal amplification, *SNR*_out_ = *β*Δ*I*_D_/(*βi*_D_+*i*_n_). Thus, when external readout noise is significant, local amplification improves the effective SNR by reducing the relative contribution of *i*_n_. Local amplification is beneficial when *i*_n_ is significant and noise form amplifier remains below the sensor noise floor, but it cannot improve the intrinsic sensor SNR when *i_D_* dominates. Larger amplifiers may reduce input‐referred noise, but at the cost of increased footprint, parasitic capacitance, power consumption, and slower response. Thus, amplifier selection must be co‐optimized with application requirements such as array density, bandwidth, dynamic range, and detection limit.

Our earlier ion‐gated bipolar amplifier (IGBA) demonstrated this concept by intimately integrating an ISFET with a vertical bipolar transistor for immediate current amplification [48] (Figure 7e). The device achieved about 70‐fold internal amplification (*β*), retained this gain during pH sensing, and suppressed external noise interference before the signal reached the external readout stage. This work established that local bipolar amplification can improve sensing performance without adding measurable LFN from the amplifier itself.

However, the vertical bipolar integration is not compatible with SiNWFETs or SiNRFETs, since they are typically fabricated on thin SOI layers. We therefore developed LBJTs on SOI as CMOS‐compatible local amplifiers [49, 50, 118] (Figure 7f,g). Standalone LBJTs showed peak current gain above 50 and input‐referred LFN much lower than that of typical SiNWFET sensors (Figure 7h,i), supporting their use as local amplifiers for nanoscale ISFETs. Substrate bias provides an additional tuning knob. A moderate positive *V*_sub_ enhances emitter‐to‐base injection and improves *β*, whereas excessive *V*_sub_ shifts the current path toward the BOX/Si interface and increases trap‐related noise. Therefore, optimal LBJT operation requires balancing current gain and interface‐noise coupling.

The latest LBJT‐NRFET device integrates a SiNRFET ion sensor and an LBJT on the same SOI substrate [50] (Figure 7g), with the SiNRFET source directly connected to the LBJT base. This architecture enables immediate amplification of the nanoribbon sensor current before exposure to external interference. The integrated device achieved a peak current gain above 20 and improved the overall SNR by 3–7 times compared with a standalone NRFET. In electrolyte pH sensing, the LBJT‐NRFET preserved the pH response while suppressing the prominent 50 Hz interference peak (Figure 7j), and the measured pH‐sensing SNR gain was about 3.1, with total noise voltage, *v*_total_, reduced from 1.95 mV for the standalone NRFET to 0.63 mV for the LBJT‐NRFET.

Overall, readout‐level correction improves array robustness, but local LBJT amplification provides a more fundamental signal‐preservation strategy which strengthens weak nanoscale sensor signals at the device level before external noise contamination. This makes LBJT‐based amplification especially relevant for high‐density ion‐sensing arrays, lab‐on‐chip systems, and low‐current nanosensors operating in electrically noisy environments. The benefit of local amplification should also be considered together with several practical design factors. In high‐density arrays, power consumption, heat generation, amplifier footprint, routing, and multiplexing architecture need to be optimized to maintain scalability. Pixel‐to‐pixel gain variation may also require calibration, especially when quantitative comparison across large arrays is needed. In addition, local amplification is most effective when downstream readout noise or environmental interference is a significant contributor to the total noise budget, while the amplifier input‐referred noise remains sufficiently low. Sufficient voltage/current operating margin and dynamic range should also be maintained, so that large pH, ion, or biomolecular responses can be amplified without saturation or signal compression. Therefore, local amplification is best viewed as a signal‐preservation strategy that should be codesigned with the sensor operating point, array architecture, power budget, and calibration scheme.

## 5 Applications Enabled by Low‐Noise Nanoscale FET Sensors

Low‐noise nanoscale FET sensors are particularly valuable for small sample volumes and in applications where the original signal approaches the assay's noise floor. As illustrated in Figure 8a (left) [139], reducing the equivalent input noise lowers the minimum resolvable surface‐potential shift and therefore improves both the SNR and the effective limit of detection (LoD). For a calibrated FET sensor, this relation can be expressed conceptually as

$$\mathrm{LoD}\approx \frac{kV_{n,\mathrm{rms}}}{S}$$

Where *V*_n,rms_ is the equivalent input voltage noise, *S* is the transduction sensitivity, and *k* is the detection criterion. However, for biomolecular assays, LoD is not determined by *V*_n,rms_ alone. Binding affinity, nonspecific adsorption, background signal, Debye screening, surface drift, and readout resolution jointly define the detectable concentration range. Therefore, low‐noise FET sensors should not be presented as universally necessary for all sensing applications. Their strongest impact appears in weak‐signal scenarios, where low‐abundance biomolecular detection [140], single‐charge interfacial events [24], single‐cell or few‐cell metabolic dynamics [141], and dense chemical imaging arrays [69] are investigated.

**FIGURE 8:**
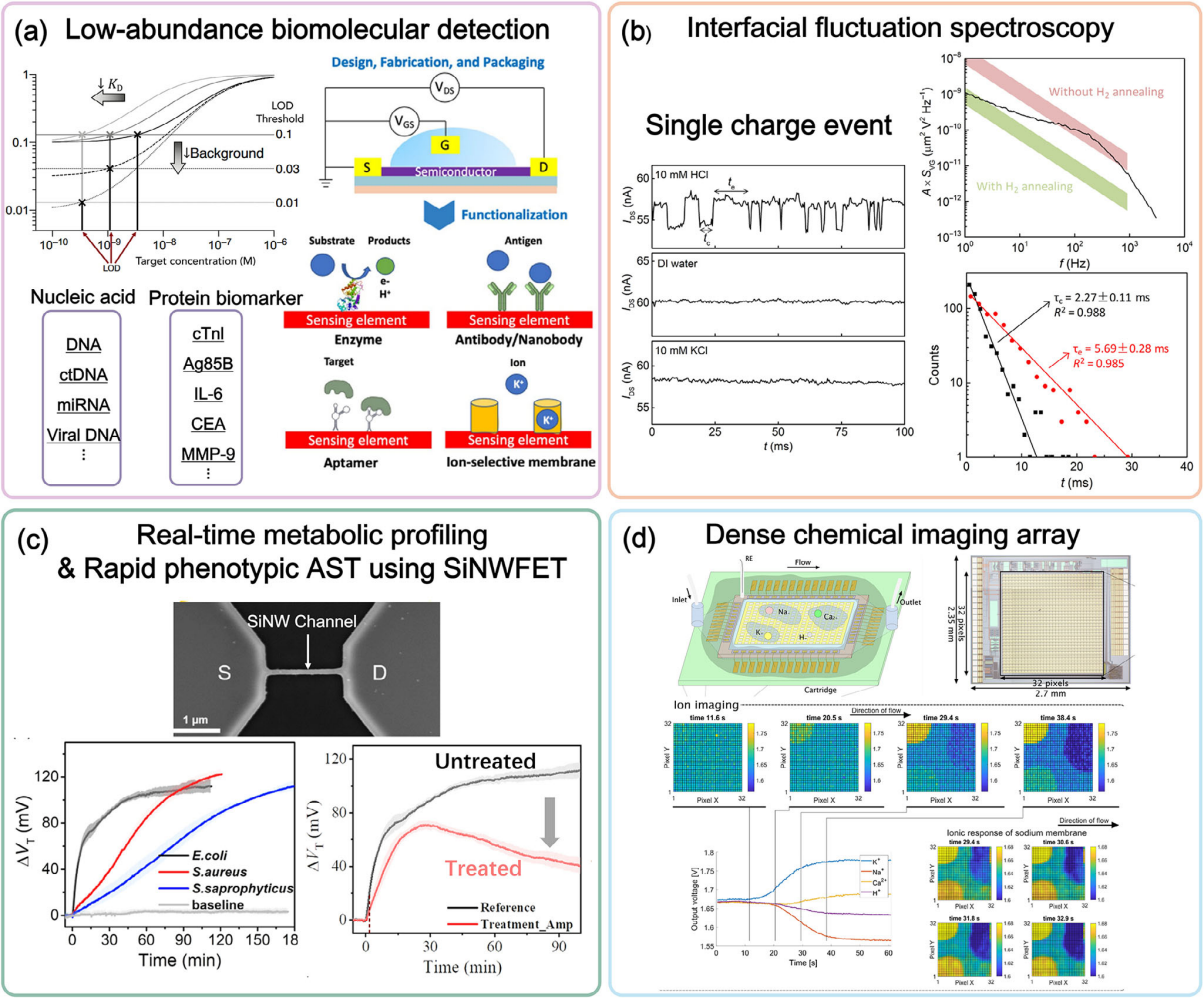
Low‐noise SiNWFET sensing for weak‐signal biochemical applications. (a) Conceptual relation between background, readout noise, binding response, and effective low detection limit (LoD) [139] (left). And schematic of low‐abundance biomolecular diagnostics, such as nucleic acid and protein biomarker detection [142] (right). (b) Interfacial fluctuation spectroscopy showing single charge trapping/detrapping events in sub‐10‐nm SiNWFET after H_2_ annealing process [24]. (c) Real‐time metabolic profiling that can distinguish different bacterial strains(bottom left) and rapid phenotypic AST within 30 min using our SiNWFET(bottom right) [68]. (d) Dense chemical imaging arrays enable mapping of weak spatial gradients and transient ionic microenvironments [69]. (Adpated with permission (a)(left) from Ref [139], copyright 2020, Elsevier; (a) (right) from Ref [142], under the terms of the CC BY, (b) from Ref [24] under the terms of the CC BY‐NC license, (c) from Ref [68] under the terms of the CC BY license, (d) from Ref [69] under the terms of the CC BY license.

In low‐abundance biomolecular diagnostics [142] (Figure 8a (right)), the target‐induced surface‐potential shift is often small because only a small fraction of receptors is occupied and the biomolecular charge is partially screened by the electrolyte [143]. SiNWFET biosensors have been widely explored for ion, metabolite, hormones, nucleic acid, and protein biomarkers detection, including ctDNA, miRNA, viral DNA, cardiac troponin, IL‐6, CEA, MMP‐9, and tuberculosis Ag85B [144, 145, 146]. Reported examples include ctDNA detection down to 10 aM using SiNW arrays [147] and miRNA detection down to 1 fM using PNA/SiNWFETs [62]. These applications motivate high‐SNR transducers because early‐stage disease biomarkers are often present at trace concentrations. The same literature [146] also emphasizes that Debye screening remains a major limitation. High ionic strength shortens the Debye length and weakens the electrostatic coupling between the target charge and the channel. Thus, low‐noise transducers must be combined with optimized surface chemistry, receptor density, low‐background interfaces, and buffer conditions to translate high electrical resolution into a lower practical LoD.

Interfacial fluctuation spectroscopy is another critical application (Figure 8b), where the objective is not only to detect analytes, but to resolve individual stochastic events at the solid–liquid interface. This is the most stringent test of low‐noise SiNWFETs. In our sub‐10‐nm electrolyte‐gated SiNWFETs [24], direct detection of single H^+^ binding and debinding events at individual Si dangling bonds was achieved only after reducing the intrinsic device noise below the single‐charge signal. Control measurements performed using the same SiNWFET in DI water and 10 mM KCl, with the drain current maintained at the same level as in 10 mM HCl, did not exhibit two‐level switching. This observation rules out conventional solid–solid interface RTS as the origin of the observed signal. Furthermore, the gate‐voltage and HCl‐concentration dependence of the switching kinetics strongly indicates that the discrete signal originates from the binding and debinding of H^+^ at an activated surface Si dangling bond. The work showed that top‐down process‐induced surface roughness and defects would otherwise obscure the Lorentzian signal from single H^+^‐surface interactions. This example clearly demonstrates the central role of low noise: without a sufficiently low intrinsic noise floor, the interfacial kinetics remain hidden beneath device fluctuation.

Low‐noise SiNWFETs are also highly relevant for single‐cell and few‐cell metabolic response monitoring via measuring metabolism‐induced extracellular pH changes, including rapid antibiotic susceptibility testing [68, 148, 149, 150, 151]. Bulk metabolism assays often generate large pH shifts and may not require ultralow noise (Figure 8c) [68]. In contrast, single‐cell or few‐bacteria measurements produce small, local, and slowly evolving ionic changes. Microfluidic hydrodynamic trapping [152] provides a route to isolate individual cells or small cell populations under controlled conditions, taking our trapping results as an example [153], avoiding population‐averaged responses and enabling analysis of cell‐to‐cell heterogeneity. The hydrodynamic trapping literature shows that microfluidics can retain cells in defined traps without external optical, magnetic, or electrical forces [154], preserving a more natural cellular state while enabling downstream analysis. When such traps are integrated with SiNWFET or ISFET arrays, low‐noise readout becomes critical for resolving weak early metabolic divergence before the response develops into a large equilibrium pH shift. Single‐bacteria/few‐bacteria testing provides information that bulk assays cannot access. Bulk measurements report population‐averaged growth or metabolism, which can mask rare but important phenotypes such as persisters, tolerant cells, or minor resistant subpopulations [155, 156]. In contrast, microfluidic trapping enables direct observation of cell‐to‐cell variability and early antibiotic‐induced metabolic changes [148, 157, 158]. Because the local pH/ion signal from one or a few bacteria is intrinsically weak, low‐noise SiNWFET/ISFET transducers are essential for resolving subtle early trajectories before they develop into large bulk endpoint signals.

Dense chemical imaging arrays represent another application class where low noise becomes increasingly important (Figure 8d) [69]. As pixel size and sensing area decrease, the number of fluctuators averaged per pixel decreases and the local chemical signal becomes more spatially heterogeneous. Large‐scale FET sensor integration has already enabled high‐density pH, ion, dopamine, and nucleic‐acid sensing platforms [159, 160, 161, 162, 163]. Such systems benefit from electronic multiplexing, redundancy, differential referencing, and fluidic integration, but they also expose pixel‐to‐pixel noise, contact variation, and local interfacial instability. Low‐noise sensors are therefore essential for transforming dense arrays from simple endpoint detectors into quantitative chemical imagers capable of resolving weak spatial gradients, transient ion dynamics, and heterogeneous biological microenvironments.

Although this review primarily focuses on electrolyte‐gated nanoscale Si FET sensors, the same LFN framework is broadly applicable to gas‐sensitive FETs and semiconductor gas sensors, where noise and drift also critically determine practical detection limits and selectivity [164]. In gas sensors, the measured LFN is often governed not only by the electronic transducer itself, but also by adsorption/desorption kinetics, surface‐trap occupation, grain‐boundary modulation, oxygen‐vacancy‐mediated charge transfer, and subgap density‐of‐states in the gas‐sensitive material [164, 165, 166]. These processes can induce slow conductance fluctuations and baseline drift, which are conceptually analogous, at the system level, to binding/debinding dynamics and dissipative interfacial‐admittance fluctuations at solid–liquid interfaces. Recent studies on oxide TFT‐type gas sensors and CMOS‐integrated gas sensors have shown that LFN analysis can reveal thermally activated excess‐noise mechanisms, assess whether a large sensing response is accompanied by a sufficiently low noise floor, and even provide gas‐selective fluctuation fingerprints through noise spectroscopy [165, 166, 167, 168]. Therefore, as in liquid‐gated biosensors, gas‐sensor performance should not be evaluated by response amplitude alone. Instead, the SNR, drift stability, fluctuation spectrum, and readout architecture should be considered together when assessing practical detection limits, selectivity, and long‐term reliability [164, 168].

## 6 Conclusion

LFN is a key limitation for nanoscale FET sensors when the target signal is weak, slow, local, or close to the background. Although these devices are often valued for high sensitivity, label‐free operation, miniaturization, and CMOS compatibility, their practical resolution is ultimately governed by the full noise and stability budget of the sensing system. This includes intrinsic FET device noise, solid–liquid interfacial fluctuations, reference‐electrode instability, sensing‐surface buffering, readout noise, and long‐term drift.

Classical LFN models, including carrier‐number fluctuation, mobility fluctuation, and correlated carrier‐number/mobility fluctuation, describe trap‐mediated noise at solid‐state interfaces. In electrolyte‐gated operation, however, the oxide/electrolyte interface introduces additional fluctuation sources. Dynamic site‐binding and impedance‐based models indicate that stochastic proton binding/debinding, interfacial admittance, and dissipative electrochemical processes can generate their own voltage noise. Therefore, once the intrinsic transistor noise is sufficiently reduced, the extrinsic solid–liquid interface may become the dominant noise source.

Noise‐reduction strategies should therefore be considered hierarchically. Gate‐stack optimization, annealing, passivation, and channel‐surface smoothing reduce trap density and process‐induced defects. Current‐path engineering, such as dual‐gate, tri‐gate, and substrate‐bias operation, weakens the coupling between remaining traps and the conductive channel. Beyond these incremental approaches, SJGFETs provide an architectural strategy for eliminating gate‐oxide/channel interface trap‐related noise by replacing the conventional oxide‐controlled gate with a metal/semiconductor Schottky junction. Our SJGFET work demonstrates that redesigning the gate‐control mechanism can push intrinsic transducer noise below conventional MOS type FET levels and approach the regime where solid–liquid interfacial noise becomes experimentally accessible.

At the same time, low transistor noise alone does not guarantee high‐performance sensing. Biomolecular FETs are often limited by Debye screening, receptor density, nonspecific adsorption, molecular‐layer drift, and surface buffering. Similarly, long‐term ISFET operation is limited by drift from hydration, charge relaxation, and ion penetration. Our AC pulse gate‐bias strategy addresses this issue by alternating sensing and recovery phases to suppress HfO_2_‐related ion‐penetration drift. Local LBJT amplification further improves system‐level SNR by strengthening weak sensor signals before external readout noise is introduced.

The most compelling applications of low‐noise SiNWFETs are therefore not large endpoint assays, but weak‐signal scenarios: low‐abundance molecular diagnostics, single‐charge or proton‐binding event detection, single‐cell or few‐bacteria metabolic analysis, early antibiotic‐response monitoring, and dense chemical imaging. Future low‐noise ISFET technologies should move from isolated device optimization toward full‐chain SNR preservation, integrating ultralow‐noise transducers, stable sensing interfaces, drift‐controlled biasing, local amplification, microfluidics, and data‐driven interpretation.

## Author Contributions

**Yingtao Yu**: writing – original draft, conceptualization. **Zheqiang Xu**: writing – review and editing. **Yihan Wang**: writing – review and editing. **Qitao Hu**: writing – review and editing, supervision. **Paul Solomon**: writing – review and editing, conceptualization, supervision. **Zhen Zhang**: conceptualization, writing – review and editing, supervision, funding acquisition.

## Conflicts of Interest

The authors declare no conflicts of interest.

## Data Availability

The data that support the findings of this study are available from the corresponding author upon reasonable request.
